# Exon junction complex shapes the m^6^A epitranscriptome

**DOI:** 10.1038/s41467-022-35643-1

**Published:** 2022-12-23

**Authors:** Xin Yang, Robinson Triboulet, Qi Liu, Erdem Sendinc, Richard I. Gregory

**Affiliations:** 1 Stem Cell Program, Division of Hematology/Oncology, Boston Children’s Hospital, Boston, MA 02115 USA; 2 Department of Biological Chemistry and Molecular Pharmacology, Harvard Medical School, Boston, MA 02115 USA; 3 Department of Pediatrics, Harvard Medical School, Boston, MA 02115 USA; 4 Harvard Stem Cell Institute, Cambridge, MA 02138 USA; 5 Harvard Initiative for RNA Medicine, Boston, MA 02115 USA; 6 Present Address: Twentyeight-Seven Therapeutics, Watertown, MA 02472 USA; 7 Present Address: Rice Research Institute, Guangdong Academy of Agricultural Sciences, Guangzhou, 510640 China; 8 Present Address: Guangdong Key Laboratory of New Technology in Rice Breeding, Guangzhou, 510640 China

**Keywords:** RNA modification, Transcriptomics

## Abstract

N6-methyladenosine (m^6^A), the most abundant modification of mRNA, is essential for normal development and dysregulation promotes cancer. m^6^A is highly enriched in the 3’ untranslated region (UTR) of a large subset of mRNAs to influence mRNA stability and/or translation. However, the mechanism responsible for the observed m^6^A distribution remains enigmatic. Here we find the exon junction complex shapes the m^6^A landscape by blocking METTL3-mediated m^6^A modification close to exon junctions within coding sequence (CDS). Depletion of EIF4A3, a core component of the EJC, causes increased METTL3 binding and m^6^A modification of short internal exons, and sites close to exon-exon junctions within mRNA. Reporter gene experiments further support the role of splicing and EIF4A3 deposition in controlling m^6^A modification via the local steric blockade of METTL3. Our results explain how characteristic patterns of m^6^A mRNA modification are established and uncover a role of the EJC in shaping the m^6^A epitranscriptome.

## Abstract (web summary)

Here the authors show the exon junction complex (EJC) component, EIF4A3, locally restricts METTL3- mediated mRNA methylation at exon junctions to explain the observed widespread enrichment of m6A modification in 3’ untranslated regions.

## Introduction

Chemical modifications of RNA, the recently-termed ‘epitranscriptome’ play key roles in many biological processes^1^. N6-methyladenosine (m^6^A) is the most abundant internal modification found in messenger RNA (mRNA)^1^. Numerous recent studies linked m^6^A to various aspects of mRNA metabolism, including splicing, localization, stability, and translation^1–11^. m^6^A is catalyzed by the methyltransferase (MTase) (writer) complex^12,13^, the core components of which are METTL3 and METTL14. Biochemical reconstitution and structural studies have demonstrated that this heterodimeric METTL3-METTL14 complex is necessary and sufficient for m^6^A modification of substrate RNAs in vitro. METTL3 is the catalytic SAM-binding subunit but requires the METTL14 co-factor that likely mediates RNA-binding^14^. However, this core enzyme interacts with numerous other proteins, including VIRMA, ZC3H13, RBM15, HAKAI, and WTAP, as part of an ~1 MDa MTase complex in cells^15,16^. The precise role of these accessory proteins is currently not well defined. METTL3/14 are essential for normal development^17–21^ and dysregulation can drive tumorigenesis and is implicated in numerous different cancer types^22^.

Transcriptome-wide mapping of m^6^A modification in different cell types using methylated RNA immunoprecipitation and sequencing (meRIP-seq), as well as more recently using antibody-independent approaches, has revealed that m^6^A occurs on large subsets of several thousand mRNAs within a particular cell, and that modification occurs at a consensus ‘RRACH’ motif (R = A/G, H = A/C/U)^23,24^. Intrinsic substrate specificity of the METTL3-METTL14 MTase can explain the preferential modification of this consensus motif, yet only about 5% of all RRACH motifs are m^6^A-modified in cellular RNA^25^. FTO and ALKBH5 have been identified as demethylases (erasers) capable of m^6^A removal^11,26^. Importantly, m^6^A is not evenly distributed throughout mRNAs and is instead highly enriched in 3’ UTRs at sites close to the stop codon, as well as within long internal exons.

Despite the major importance of m^6^A in post-transcriptional gene control, the mechanisms responsible for its distribution throughout the transcriptome remain largely unknown. m^6^A modification is considered a co-transcriptional event^27–30^, and it is therefore expected that m^6^A profiles are established in nascent RNA. However, the distribution of m^6^A in nascent RNA is controversial. Unlike mature mRNAs, m^6^A methylation is not restricted to the 3’ UTR of nascent RNA transcripts^29,31,32^. Ke et al. found ~93% of m^6^As in chromatin-associated RNA to be located in exons^27^. Using TNT-seq to specifically examine m^6^A in newly synthesized RNAs, however, Louloupi et al. found most m^6^A sites to be located in introns^28^. Both studies found more m^6^A near splice junctions in nascent RNA compared with mature RNA, suggesting that mRNA maturation can reshape the m^6^A topology. So far, studies have implicated MTase recruitment by polymerase II and trans-acting factors, including transcription factors, RNA-binding proteins, or histone modifications, to promote localized m^6^A modification^4,33–37^. For example, it has been proposed that H3K36me3 chromatin modification might help recruit METTL3-METTL14 and thereby enrich m^6^A towards the 3’-end of mRNAs^37^. In addition, non-coding RNAs are involved in m^6^A modification deposition by recruiting m^6^A regulators to the UTR region^38^. Increasing evidence indicates that m^6^A demethylases associate with chromatin and engage in RNA splicing regulation^29,30,39,40^. However, none of these known links can adequately explain the characteristic m^6^A distribution across mRNAs with a strong peak in the 3’ UTR, and enrichment of m^6^A in long internal exons.

In this study, we explore the mechanism responsible for the characteristic m^6^A profile that has been observed for over a decade. We find the core component of the exon junction complex, EIF4A3, shapes the m^6^A landscape by blocking METTL3-mediated m^6^A modification close to exon-exon boundaries within the mRNA coding sequence (CDS). Our conclusions are based on METTL3 eCLIP-seq and m^6^A meRIP-seq data showing that METTL3 binding and m^6^A modification is restricted by EIF4A3, in particular within short internal exons and in the vicinity of exon-exon boundaries. We provide additional evidence using reporter gene assays to further support this conclusion. Our results explain how m^6^A topology is established and uncover a role of the EJC in shaping the m^6^A epitranscriptome.

## Results

### Dynamic m^6^A profiles during mRNA maturation

**Fig. 1:**
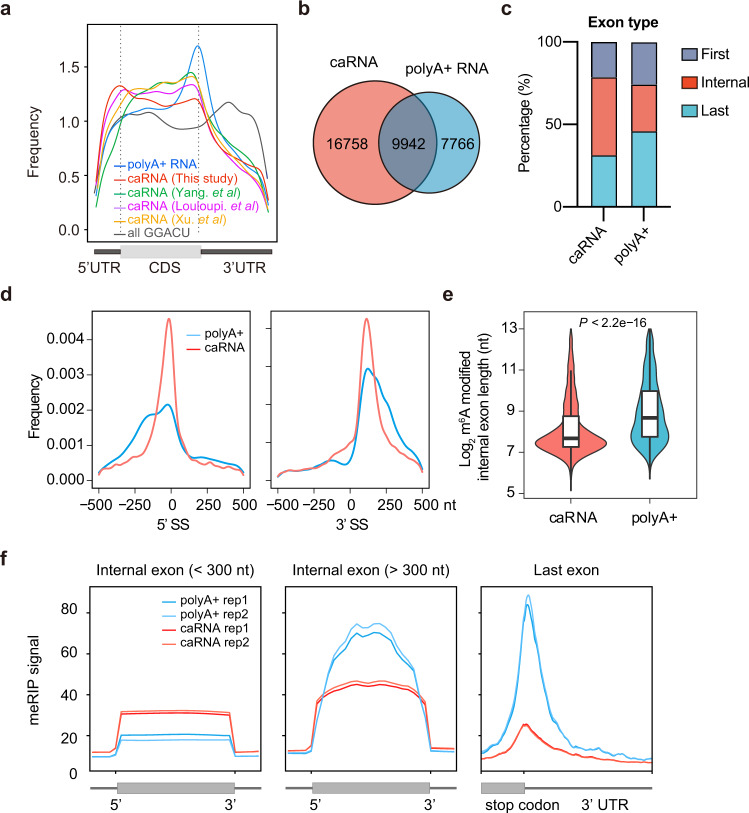
Nascent transcripts contain more m^6^A in short internal exons compared with mature mRNAs. **a** Metaplot of meRIP-Seq showing the m^6^A distribution in polyA+ RNAs compared with chromatin-associated RNAs (caRNAs). Also shown is the distribution of the GGACU sequence motif. **b** Venn diagram showing the overlap of m^6^A peaks identified in polyA+ RNAs and caRNAs. **c** Barplot showing the percentage of m^6^A peaks within the first, internal, and last exons of protein-coding genes. **d** Distribution of the distance of m^6^A peaks to the closest splice site. **e** The length of internal exons with m^6^A peaks identified in caRNA, polyA+ RNA. The three lines inside the violin plots are the first quartile, median, and third quartile. *n* = 9646; 3392. Statistical analyses, unpaired two-tailed Student’s *t*-test. **f** Aggregation plots of meRIP-Seq read signals in short-, or long- internal exons, and last exon. Internal exons were divided into two groups based on length.

To begin to explore the mechanism(s) responsible for the characteristic m^6^A distribution on mature mRNAs, we isolated chromatin-associated RNA (caRNA; rRNA depleted and enriched for nascent transcripts), and polyA+ RNA (mature mRNA) from HeLa cells, and quantified m^6^A modification levels by HPLC-MS/MS. Global m^6^A levels were found to be similar between caRNA and polyA+ RNA (Supplementary Fig. 1a). Considering the presence of other types of non-coding RNA in the caRNA fraction that could influence global m^6^A measurements, we performed m^6^A methylated RNA immunoprecipitation sequencing (MeRIP-seq), and compared m^6^A modification profiles between caRNA and polyA+ mRNA. As expected, although the RRACH motif is quite evenly distributed across mRNAs (Fig. 1a), a strong peak of m^6^A close to the stop codon was observed in mature mRNAs (Fig. 1a). Distinct, however, from this characteristic m^6^A modification landscape in polyA+ RNA, m^6^A modification in caRNA was much more evenly distributed (Fig. 1a), a finding that is consistent with other publicly available MeRIP-seq datasets^2,28,29^. Among the m^6^A peaks located in exons of protein-coding genes, 16,758 and 7766 peaks were exclusively detected in caRNAs or polyA+ RNAs, respectively, whereas 9942 peaks are common to both RNA samples (Fig. 1b). m^6^A occurred at the consensus motif in both polyA+ and caRNA (Supplementary Fig. 1b), and mainly within exons of protein-coding genes (Supplementary Fig. 1c). Notably, the m^6^A peaks in caRNA are mostly located in internal exons, whereas in polyA+ RNA the m^6^A peaks are strongly enriched in last exons (Fig. 1c). Furthermore, polyA+ RNA had higher overall m^6^A modification than caRNA (Supplementary Fig. 1d). More detailed analysis showed relative enrichment of m^6^A close to splice sites in caRNA compared to polyA+ RNA (Fig. 1d). Moreover, the length of internal exons with specific m^6^A peaks in caRNAs was generally shorter than those of polyA+ RNAs (Fig. 1e). We then divided internal exons into groups based on their length and found that the m^6^A signal in short internal exons of caRNAs to be higher than in polyA+ RNAs. An opposite pattern was observed for long exons and regions near the stop codon with a higher m^6^A signal in polyA+ RNA (Fig. 1f). Together, these results support dynamic patterns of m^6^A modification during mRNA maturation, with relatively more m^6^A present in short internal exons of nascent mRNAs compared with mature mRNAs. These observations raise the possibility that m^6^A may be actively removed from regions close to exon-exon boundaries during mRNA maturation, and/or extra m^6^A is deposited more distal to exon-exon boundaries in spliced mRNAs (Supplementary Fig. 1e).

### Minimal role of ALKBH5 demethylase during mRNA maturation

Since m^6^A demethylases (FTO and ALKBH5) have been reported to be involved in pre-mRNA splicing regulation^11,40,41^, we first hypothesized that dynamic m^6^A distribution during mRNA maturation might involve removal of m^6^A in internal exons by FTO or ALKBH5 accompanied by splicing factors. To explore this, we performed pull-down assays coupled with protein mass spectrometry and found ALKBH5, but not FTO, interacts with numerous splicing factors (Supplementary Fig. 2a, b and Supplementary Data 1). Further analysis showed components of the Exon Junction Complex to be most highly enriched, and we verified this by immunoprecipitation and Western blots (Supplementary Fig. 2c, d and Supplementary Table 1). EJC is a multiprotein complex that deposited ~24 nucleotides upstream of exon-exon junctions during mRNA maturation. EJC assembly begins during splicing, and the first step involves the position-specific deposition of the DEAD-box protein EIF4A3 onto RNA by the spliceosome^42,43^. We therefore next examined the role of ALKBH5, and the core component of the EJC, EIF4A3, in modulating the m^6^A epitranscriptome. We individually knocked down ALKBH5 and EIF4A3 using siRNAs and measured global changes in m^6^A levels of polyA+ RNA by HPLC-MS/MS. We found ALKBH5 depletion had a very minor effect on m^6^A levels. Strikingly, however, EIF4A3 depletion dramatically increased the global m^6^A level in polyA+ RNA (Supplementary Fig. 2e, f). These results pointed to a possible role of EIF4A3 and the EJC in shaping the m^6^A epitranscriptome.

### The core EJC subunit EIF4A3 influences m^6^A profiles

**Fig. 2:**
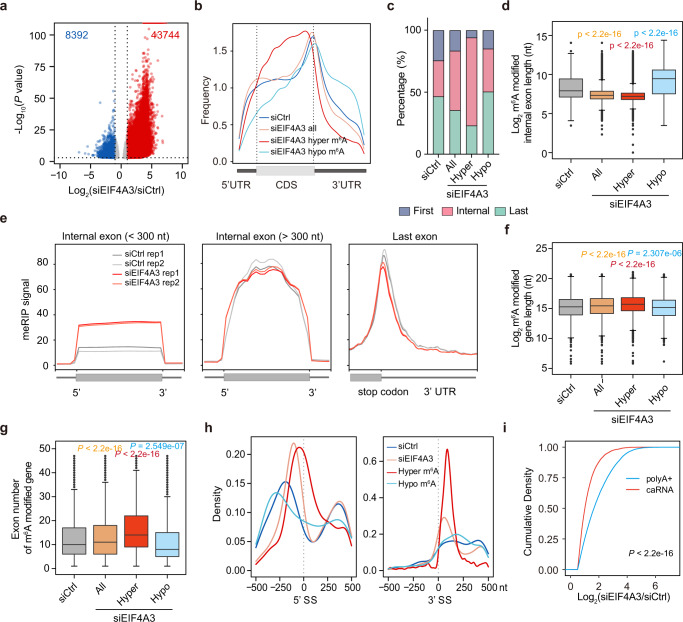
Depletion of EIF4A3 increases m^6^A modification of short internal exons. **a** Differential m^6^A regions upon EIF4A3 depletion. Red dots represent increased m^6^A regions (hypermethylated m^6^A), Blue dots represent decreased m^6^A regions (hypomethylated m^6^A). **b** Metaplot showing the distribution of all m^6^A peaks in siCTRL and siEIF4A3, hypermethylated, hypomethylated m^6^A. **c** Barplot showing the percentage of different exons with m^6^A peaks in siCTRL and siEIF4A3, hypermethylated, hypomethylated m^6^A. **d** The length of m^6^A modified internal exons in siCTRL and siEIF4A3, and the length of internal exons with hypermethylated or hypomethylated m^6^A. Solid line represents median, with whiskers indicating minimum to maximum values. *n* = 1743; 6643; 44,010; and 1854. Statistical analyses, unpaired two-tailed Student’s *t*-test. **e** Aggregation plots showing m^6^A enrichment in short, long and last exon. Internal exons were divided into two groups based on length. **f** The length of m^6^A modified genes in siCTRL and siEIF4A3, and the length of genes with hypermethylated or hypomethylated m^6^A. Solid line represents median, with whiskers indicating minimum to maximum values. *n* = 10,335,; 12,291; 44,293; and 8238. Statistical analyses, unpaired two-tailed Student’s *t*-test. **g** The exon number of m^6^A modified genes in siCTRL and siEIF4A3, and the exon number of genes with hypermethylated or hypomethylated m^6^A. Solid line represents median, with whiskers indicating minimum to maximum values. *n* = 10,335; 12,291; 44,293; and 8238. Statistical analyses, unpaired two-tailed Student’s *t*-test. **h** Distribution of the distance of m^6^A peak summits to the closest splicing sites. **i** Cumulative curves showing fold-change of hypermethylated m^6^A induced by EIF4A3 KD in polyA+ RNA and caRNA. *P* value was calculated using two-sided Wilcoxon and Mann–Whitney test.

Based on our findings that EIF4A3- but not ALKBH5-depletion impacts global m^6^A levels, we next explored how the m^6^A epitranscriptome might be influenced by EIF4A3 and considered an alternative model whereby the methylation of internal exons might be hindered by the EJC rather than requiring ALKBH5-mediated demethylation of these sites (Supplementary Fig. 1e). In order to identify differentially modified sites more comprehensively, we utilized RADAR^44^. Consistent with our HPLC-MS/MS results showing substantially increased global m^6^A level in polyA+ RNA in EIF4A3-depleted cells, MeRIP-seq results showed a remarkable induction of m^6^A modification sites upon EIF4A3- but not ALKBH5-depletion (Fig. 2a and Supplementary Fig. 2g, h). Further analysis revealed that EIF4A3 KD caused 43,744 and 8392 regions to be hypermethylated and hypomethylated, respectively (Fig. 2a), with most hypermethylated and hypomethylated m^6^A regions annotated to protein-coding genes (Supplementary Fig. 3a). Metaplot showed that hypermethylated regions upon EIF4A3 depletion are highly enriched in the CDS, while the pattern of hypomethylated sites was similar to overall m^6^A (Fig. 2b). Considering previous studies showing enrichment of EIF4A3 binding within short internal exons of spliced mRNAs^43,45^, we next explored whether exons with hypermethylated m^6^A upon EIF4A3 depletion have similar features. Indeed, we found the proportion of internal exons with m^6^A modification was increased in EIF4A3-depleted cells, with the hypermethylated m^6^A sites being highly enriched in internal exons (Fig. 2c). We next analyzed the relative length of internal exons with altered m^6^A modification upon EIF4A3 knockdown and found a strong enrichment of hypermethylated sites localized within short internal exons (Fig. 2d). EIF4A3 depletion significantly increased m^6^A modification of short internal exons, but not long exons or the 3’ UTR (Fig. 2e). For long exons, although the m^6^A signal was overall unchanged upon EIF4A3 knockdown, increased m^6^A was specifically observed at sites proximal to splice junctions (Supplementary Fig. 3b). Moreover, we found that hypermethylated m^6^A sites upon EIF4A3 depletion are enriched in longer mRNAs containing more exons (Fig. 2f, g), which is highly consistent with known features of EJC deposition^45,46^. In addition, we analyzed the methylation status of single-exon (intronless) genes, that are expected to be substantially less or not associated with the EJC^43,45,47^. We found that single-exon genes have a higher m^6^A level than the internal exons of multi-exon genes (Supplementary Fig. 3c), and that depletion of EIF4A3 did not impact m^6^A peak enrichment of single-exon genes (Supplementary Fig. 3d). Next, considering the relative enrichment of m^6^A close to splice sites in caRNA compared to polyA+ RNA (Fig. 1d), as well as that the EJC resides ~24 nucleotides upstream of exon-exon junctions^43,45,46,48^, we next analyzed changes in m^6^A modification due to EIF4A3 deficiency at splice sites. EIF4A3 KD resulted in increased m^6^A at splice sites (Fig. 2h), suggesting changes in m^6^A could be linked to splicing. Further analysis identified a relatively small number of changes in exon usage, and that altered exon usage is positively correlated with changes in m^6^A modification upon EIF4A3 depletion (Supplementary Fig. 3e, f). Hypermethylated m^6^A sites might be recognized by certain m^6^A reader proteins to promote exon inclusion^5,49^. Like protein-coding genes, long non-coding RNAs (lncRNAs) are transcribed by RNA polymerase II and have various lengths and exon numbers. The introns of lncRNAs are also spliced during transcription utilizing mechanisms shared with protein-coding genes. We, therefore, analyzed whether EIF4A3 influences the m^6^A modification of lncRNAs. We found 1155 hypermethylated and 669 hypomethylated regions on lncRNAs upon EIF4A3 depletion (Supplementary Fig. 4a), and like protein-coding genes, hypermethylated regions are enriched in longer genes with more exons (Supplementary Fig. 4b, c). These findings indicate that EJC broadly influences m^6^A modification of short internal exons of polymerase II transcribed RNAs. In addition, we found that EIF4A3 depletion caused a more pronounced increase in m^6^A levels on polyA+ RNAs than on caRNAs (Fig. 2i). Collectively, our data support that EIF4A3, the core RNA-binding component of the EJC, influences m^6^A modification close to splice sites within the CDS of mRNAs to shape m^6^A distribution throughout the transcriptome during mRNA maturation.

### EIF4A3 locally inhibits METTL3 binding and m^6^A modification

**Fig. 3:**
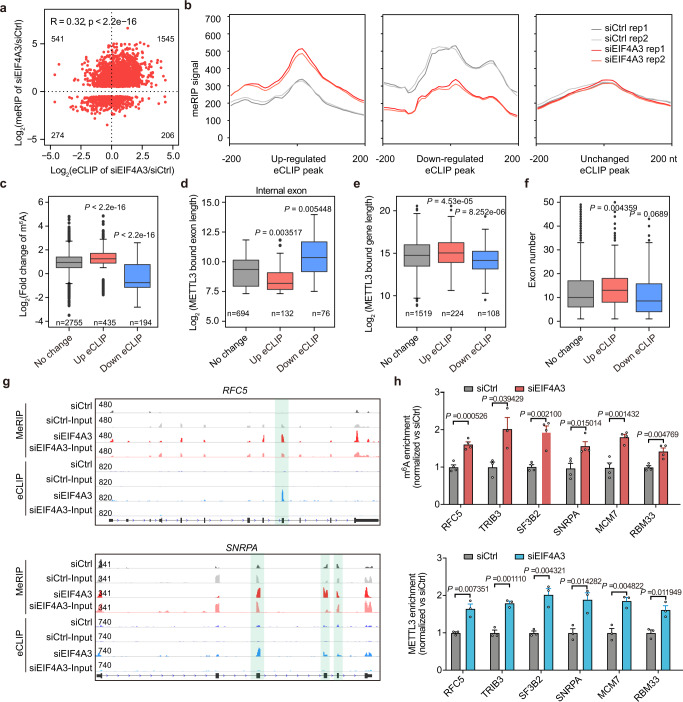
EIF4A3 locally inhibits METTL3 binding and m^6^A modification. **a** Correlation between m^6^A fold change and METTL3 binding fold change upon EIF4A3 knockdown. Correlation coefficient (r) and *P* value was calculated by Pearson’s correlation analysis. **b** Average distribution of MeRIP-seq signal is shown, aligned around increased, decreased, and unchanged METTL3 binding peaks. **c** Boxplot showing the fold change of m^6^A enrichment on METTL3 binding peaks upon EIF4A3 depletion. METTL3 binding peaks are divided into three groups: no change, up regulated and down regulated upon EIF4A3 KD. Solid line represents median, with whiskers indicating minimum to maximum values. Statistical analyses, unpaired two-tailed Student’s *t*-test. **d** The length of internal exons with unchanged, increased or decreased METTL3 binding peaks. Solid line represents median, with whiskers indicating minimum to maximum values. Statistical analyses, unpaired two-tailed Student’s *t*-test. **e** The length of protein coding genes with unchanged, increased or decreased METTL3 binding peaks. The solid line represents median, with whiskers indicating minimum to maximum values. Statistical analyses, unpaired two-tailed Student’s *t*-test. **f** The exon number of genes with unchanged, increased, or decreased METTL3 binding peaks. Solid line represents median, with whiskers indicating minimum to maximum values. *n*  =  1519; 224; 108. Statistical analyses, unpaired two-tailed Student’s *t*-test. **g** Genome Browser tracks of meRIP-seq and METTL3 eCLIP-seq read coverage at gene *RFC5* and *SNRPA* in siCtrl and siEIF4A3 HeLa cells. **h** meRIP-qPCR and METTL3 CLIP-qPCR analysis showing increased m^6^A modification and METTL3 binding ability upon EIF4A3 KD. Data are mean ± S.E.M. of three or four independent experiments. Statistical analyses, two-tailed Student’s t-test. Source data are provided as a Source Data file.

We postulated that EIF4A3 bound to exon-exon boundaries might locally occlude METTL3 mRNA association to shape the m^6^A epitranscriptome. To test this, we performed METTL3 eCLIP-seq in control and EIF4A3 depleted HeLa cells (Supplementary Fig. 5a–d). Further analysis identified 472 peaks from 400 genes that were significantly increased upon EIF4A3 depletion, while 231 peaks from 192 genes were decreased (Supplementary Fig. 5e). Importantly, a positive correlation was found between mRNAs with increased METTL3 occupancy and increased m^6^A modification upon EIF4A3 KD compared with control cells (Fig. 3a). Consistently, increased m^6^A modification was observed at sites with increased, but not decreased or unchanged, METTL3 occupancy upon EIF4A3 depletion (Fig. 3b, c), and METTL3 occupancy was increased at sites of increased m^6^A modification when EIF4A3 was diminished (Supplementary Fig. 5f). Furthermore, 314 of the 472 increased METTL3 binding peaks upon EIF4A3 knockdown contained hypermethylated m^6^A regions (Supplementary Fig. 5g). A highly significant correlation between sites of hypermethylation and increased METTL3 binding upon EIF4A3 knockdown was observed (Supplementary Fig. 5h). Consistent with the dynamic changes in m^6^A profiles during mRNA maturation (Fig. 1) and with the changes in m^6^A profiles we identified with EIF4A3 depletion (Fig. 2), we found that EIF4A3 KD led to specifically increased METTL3 binding to short internal exons and longer mRNAs containing more exons (Fig. 3d–f). Genome Browser tracks of MeRIP-seq and METTL3 eCLIP-seq at selected genes are shown to exemplify the role of EIF4A3 in restricting METTL3 binding near splice site junctions to influence patterns of m^6^A mRNA modification (Fig. 3g), findings that we confirmed by m^6^A meRIP-qPCR and METTL3 CLIP-qPCR analysis for several individual genes that we identified with hypermethylated m^6^A and increased METTL3 occupancy upon EIF4A3 knockdown (Fig. 3h). These findings suggest that METTL3 recruitment to spliced mRNAs is hindered by the EJC complex, resulting in lower m^6^A modification within the CDS of mRNAs and in particular in short internal exons.

### EJC blocks METTL3-mediated m^6^A modification during mRNA splicing

**Fig. 4:**
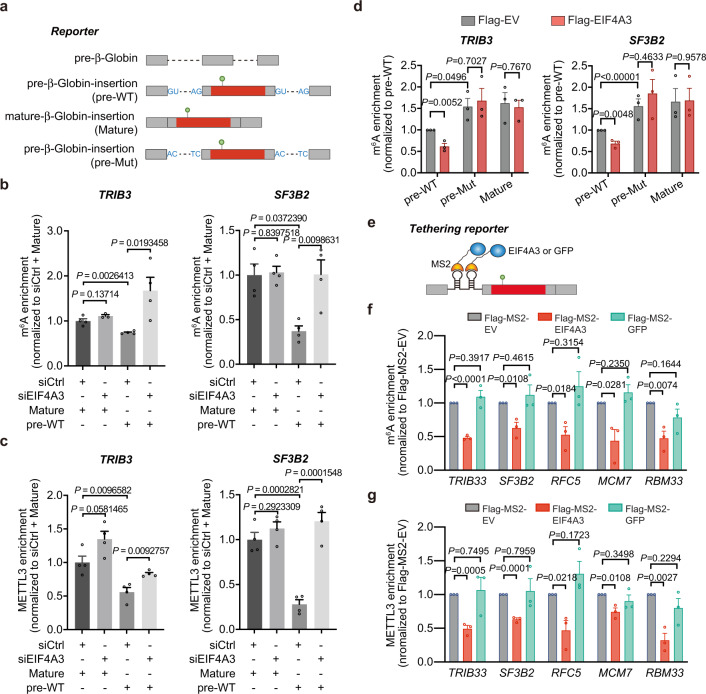
EIF4A3 blocks METTL3-mediated m^6^A modification during mRNA maturation. **a** Schematic of reporter constructs. Grey boxes and dash lines present the exons and introns of mouse beta-globin, respectively. Red boxes present the exons of candidate genes with hypermethylated m^6^A (green dot). GU/AG splicing mutation is indicated. **b**, **c** meRIP-qPCR (**b**) and METTL3 CLIP-qPCR (**c**) analysis of reporter constructs showing m^6^A and METTL3 enrichment upon EIF4A3 KD. Data are mean ± S.E.M. of three independent experiments. Statistical analyses, two-tailed Student’s t-test. **d** meRIP-qPCR analysis of pre-WT, pre-Mut, and Mature reporter constructs showing m^6^A enrichment in Flag-tagged empty vector and EIF4A3 overexpressed HeLa cells. Data are mean ± S.E.M. of three or four independent experiments. Statistical analyses, two-tailed Student’s *t*-test. **e** Schematic of tethering reporter construct. **f**,** g** meRIP-qPCR (**f**) and CLIP-qPCR (**g**) analysis of tethering reporter constructs showing decreased m^6^A enrichment and METTL3 binding ability upon MS2-EIF4A3 but not MS-GFP overexpression. Data are mean ± S.E.M. of three independent experiments. Statistical analyses, two-tailed Student’s t-test. Source data are provided as a Source Data file.

EIF4A3 is recruited to mRNA by the spliceosome during splicing and serves as a platform for the assembly of the EJC after exon ligation^50^. We reasoned that splicing is required for EJC recruitment to locally restrict METTL3 accessibility and m^6^A mRNA modification. To further test this, we employed reporter assays. We individually cloned the exonic regions of several genes we found to be both m^6^A hypermethylated and with increased METTL3 binding in EIF4A3-deficient cells, into exon 2 of the intron-containing mouse beta-Globin gene (precursor reporters, pre-WT). As controls for each gene, a mature version of beta-Globin reporter without introns but containing the same exonic inserts was generated (mature reporters, Mature) (Fig. 4a). Cells were transfected with the different reporters, and relative levels of m^6^A modification or METTL3-association was measured by α-m^6^A meRIP-qPCR or α-METTL3 CLIP-qPCR, respectively. Compared with mature reporters, precursor reporters had overall lower m^6^A levels and decreased association with METTL3 (Fig. 4b, c and Supplementary Fig. 6a, b). Moreover, EIF4A3 depletion caused substantially increased m^6^A modification and METTL3 binding to the precursor reporters, but not to the mature reporters (Fig. 4b, c and Supplementary Fig. 6a, b). We further generated specific GU/AG splicing mutant reporters (pre-Mut) and found splicing deficiency led to increased m^6^A levels compared to the corresponding wild-type pre-reporters (pre-WT) (Fig. 4a, d and Supplementary Fig. 6c). Ectopic expression of EIF4A3 decreased m^6^A levels of pre-WT but not pre-Mut or mature reporters (Fig. 4d and Supplementary Fig. 6d). Together, these results directly support our model that EJC deposition during mRNA splicing inhibits METTL3 binding and m^6^A modification.

To further directly test the role of EIF4A3 in locally blocking m^6^A deposition, we performed tethering experiments using mature mRNA reporters containing two MS2-binding sites located in the exon junction regions together with ectopic expression of an MS2-EIF4A3 or MS2-GFP fusion proteins (Fig. 4e and Supplementary Fig. 6e). We found that directly tethering MS2-EIF4A3, but not MS2-GFP control, to the exon junction region suppressed the m^6^A modification level and METTL3 binding of several different gene reporters (Fig. 4f, g). In addition, the binding capacity of the m^6^A MTase complex component WTAP to the reporter transcripts was also found to be inhibited by increased EIF4A3 expression (Supplementary Fig. 6f). These findings further directly support that the EJC, and in particular EIF4A3, blocks METTL3-mediated m^6^A modification within the vicinity of exon-exon boundaries of certain intron-containing mRNAs to shape m^6^A distribution throughout the epitranscriptome.

## Discussion

**Fig. 5:**
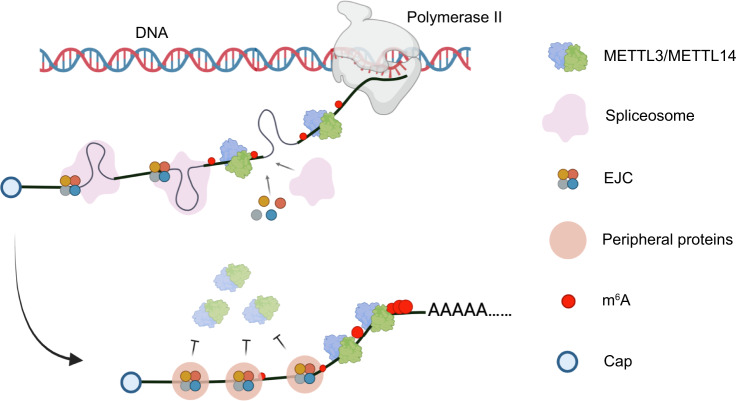
Blockade of METTL3 by the exon junction complex shapes the m^6^A epitranscriptome. Model for how the EJC locally controls METTL3-mediated m^6^A modification during mRNA processing. Created with BioRender.com.

m^6^A is highly enriched in the 3’ UTR of a large subset of mature mRNAs to influence mRNA stability and/or translation yet the mechanism responsible for the observed m^6^A distribution has remained enigmatic. In this study, we find the exon junction complex shapes the m^6^A landscape by blocking METTL3-mediated m^6^A modification close to exon junctions within the mRNA coding sequence (CDS). We propose a model in which EJC-binding at exon-exon boundaries reduces the local accessibility of the mRNA to METTL3 resulting in less m^6^A modification throughout the CDS region of mRNA. In contrast, EJC-depleted regions, including long internal exons, and terminal exons that typically comprise 3’ UTRs, are accessible to METTL3 binding and m^6^A modification (Fig. 5).

Our model is supported by METTL3 eCLIP-seq and m^6^A meRIP-seq data showing that METTL3 binding and m^6^A modification is restricted by EIF4A3. Upon EIF4A3 knockdown, m^6^A levels globally increased (measured by mass spec), and at the individual gene level positively correlated with increased m^6^A modification and METTL3 binding to mRNA. Both hypermethylated m^6^A and increased METTL3 binding regions are enriched in short internal exons, suggesting EIF4A3 blocks METTL3 from binding to short exons to deposit m^6^A. Known features of EJC occupancy across the transcriptome help further support our model of localized EIF4A3-mediated suppression of m^6^A mRNA modification. We provide additional evidence using reporter gene assays to further support this conclusion. Although EIF4A3, but not GFP, hinders m^6^A deposition, it remains possible that other RBPs might also influence m^6^A modification by a similar steric hinderance mechanism. Although we initially identified EJC components as ALKBH5-interacting proteins, we did not find any evidence for a requirement of ALKBH5 in controlling global m^6^A levels or influencing m^6^A profiles during mRNA maturation. Thus, demethylation of internal exons is unlikely to be responsible for the dynamic profile of m^6^A distribution. Therefore the relevance of the interaction between ALKBH5 and the EJC currently remains unknown. Furthermore, closer investigation reveals the apparent caRNA-specific peaks that might be expected to be removed during mRNA maturation (Fig.1b) are actually also typically modified in mature polyA+ mRNAs but are not routinely computationally identified using the standard bioinformatics tools since these sites are considerably less prominent than the more heavily modified 3’ UTR peaks that exist in mature mRNAs (Fig. 1f and Supplementary Fig. 1d). Thus, we propose that m^6^A occurs throughout the mRNA during transcription albeit at a relatively low stoichiometry, and that subsequent and locally restricted METTL3/14 activity by the EJC leads to accumulation of m^6^A in the 3’ UTR of mature mRNAs.

While our findings provide a clear molecular explanation for the long-standing question of how characteristic m^6^A profiles are established, it remains unclear why this particular m^6^A topology is needed. A previous study suggested that elevated m^6^A modifications located in CDS regions reduced translational efficiency^34^. EJC-mediated blocking of m^6^A within ORFs may function as a protective mechanism against aberrant mRNA hypermethylation in the CDS region, thereby promoting translation. The exon density and exon lengths within individual mRNAs might be an important determinant of levels of gene expression through this mechanism, with implications for the design of cDNA expression constructs both in the research lab and potentially also for gene therapies in the clinic. Alternatively, given our previous findings that METTL3 at sites close to the stop codon facilitates mRNA circularization for ribosome recycling and enhanced mRNA translation, this EJC-mediated mechanism controlling METTL3 binding and m^6^A distribution might be important to prevent alternative and non-productive mRNA looping at sites within the CDS^3^. Moreover, considering the well-established role of the EJC, it will be interesting also to explore how m^6^A modification might be related to nonsense-mediated decay (NMD) pathways.

## Methods

### Cell culture and antibodies

HeLa (ATCC,CCL-2) and HEK293T (ATCC, CRL-3216) were cultured in Dulbecco’s Modified Eagle Medium (DMEM) (Gibco, 11965-092) supplemented with 10% fetal bovine serum (FBS) (Gemini Bio., 100-106), 1 mM sodium pyruvate (Gibco, 11360070), and 0.5% penicillin/streptomycin (ThermoFisher, 15140-122) at 37 °C in a humidified incubator with 5% CO_2_. Cell lines were authenticated with morphology, karyotyping, and PCR-based approaches by ATCC.

The following antibodies were purchased from the indicated suppliers. Mouse anti-Flag M2 (sigma, F3165, 1:2000), anti-EIF4A3 (Abcam, ab32485, 1:2000), anti-Y14 (Abcam, ab5828, 1:2000), anti-MAGOH (Abcam, ab186431, 1:2000), anti-PNN (Abcam, 244250, 1:2000), anti-ALKBH5 (Cell Signaling Technology, 80283, 1:1000), anti-ALYREF (Abcam, ab202894, 1:2000), anti-METTL3 (Abcam, ab195352, 1:2000 for West blotting and 1:100 for Immunoprecipitation), anti-WTAP (Proteintech, 60188, 1:2000 for West blotting and 1:100 for Immunoprecipitation), anti-m^6^A (Abcam, ab151230, 1:100), anti-beta-Actin (Abcam, 8226, 1:3000), anti-rabbit IgG, HRP-linked Antibody (Cell Signaling Technology, 7074, 1:3000), anti-mouse IgG, HRP-linked Antibody (Cell Signaling Technology, 7076, 1:3000).

### PolyA + RNA purification

75 μg of total RNA isolated using Trizol reagent were subjected to polyA+ RNA purification. Total RNA was denatured at 65 °C for 5 min, followed by standing on ice for 2 min. 200 µl of oligo(dT)_25_ magnetic beads (NEB, S1419S) were washed with 2 × binding buffer (20 mM Tris-Cl, pH 7.5, 1 M LiCl, 2 mM EDTA), and resuspended with 100 ml of 2 × binding buffer. Denatured total RNAs were mixed with washed beads, and incubated for 15 min at R.T. with rotation. After discarding the unbound RNA, beads were washed twice with washing buffer (10 mM Tris-Cl, pH 7.5, 200 mM LiCl, 1 mM EDTA). Bound RNAs were eluted from beads with 100 µl of TE buffer (5 mM Tris-Cl, pH 7.5, 1 mM EDTA). Binding and washing steps were repeated one more time, and elute the RNAs with 20 μl of TE buffer.

### Chromatin associated RNA (caRNA) extraction

Cells were lysed with cell lysis buffer (10 mM Tris-HCl, pH7.5, 150 mM NaCl, 0.05% NP-40 and proteinase inhibitor cocktail (Roche, 11836170001)) and incubated on ice for 10 min. The suspension was carefully added at the top of sucrose buffer (10 mM Tris-HCl, pH7.5, 150 mM NaCl, 24% sucrose), and centrifuged at 3200 × *g* for 10 min. Nuclear pellets were resuspended in Glycerol buffer (20 mM Tris-HCl, pH7.5, 75 mM NaCl, 0.5 mM EDTA, pH8.0, 50% glycerol), and mixed with same volume nuclear lysis buffer (10 mM Tris-HCl, pH7.5, 300 mM NaCl, 7.5 mM MgCl_2_, 0.2 mM EDTA, pH8.0, 1% NP-40, 1 M urea) on ice for 2 min. The lysate was centrifuged at 13,000 × *g* for 2 min to precipitate the chromatin-RNA complex. The chromatin pellets were briefly rinsed with PBS-EDTA. RNA was extracted with TRIzol reagent (Invitrogen, 15596018). Ribosomal RNAs were removed using RiboMinus™ Eukaryote System v2 (ThermoFisher, A15026).

### HPLC-MS/MS analysis

500 ng RNA was digested with 100 U S1 nuclease (ThermoFisher, EN0321) at 37 °C for 2 h and dephosphorylated with 1 U rSAP (NEB # M0371S) at 37 °C for 1 h. The 100 μl samples were filtered with Millex-GV 0.22 u filters (Millipore Sigma # SLGV033RS). A total of 5–10 μl from each sample was injected into the Agilent 6470 Triple Quad LC/MS instrument with Agilent Zorbax Eclipse C18 reverse phase HPLC column. The samples were run at 500 μl/min flow rate in mobile phase buffer A (water with 0.1% Formic Acid) and 0–20% gradient of buffer B (methanol with 0.1% formic acid). MRM transitions are measured for N6-methyl adenosine (282.1–150.1), and adenosine (268.1–136.1). Standard compounds for m^6^A (Cayman Chemical #16111) were run on HPLC-MS/MS to optimize HPLC method and determine retention times for each nucleoside. For HPLC-MS/MS data collection and analysis, Agilent Mass Hunter LC/MS Data Acquisition Version B.08.00 and Quantitative Analysis Version B.07.01 software was used.

### Plasmid construction and transfection

Human FTO, ALKBH5, EIF4A3, CASC3, MAGOH, Y14, and ALYREF were cloned into pFlag-CMV2 vector. Mouse beta-Globin gene with/without introns was cloned into pBi-luciferase vectors. Exons with hypermethylated m^6^A of selected genes were inserted into second exons of beta-Globin. 2× MS2 binding site sequence was PCR-amplified from FLuc-MS2bs and inserted into the exon1-exon2 junction region of mature beta-Globin. Splicing signals GU-AG in introns of beta-Globin were mutated into AC-TC to generate pre-Mut constructs. MS2 coat protein sequence was inserted into the N terminal of EIF4A3 or GFP to generate pFlag-CMV2-MS2-EIF4A3 or pFlag-CMV2-MS2-GFP. Plasmids were transfected using Lipofectamine 2000 transfection reagent (Invitrogen, 1166809). The siRNA duplexes (Dharmacon) were transfected into cells at a final concentration of 50 nM using Lipofectamine RNAiMax (Invitrogen, 13778150) following the manufacturer’s instructions. Cells were harvested 48 h after transfection. All primers and siRNAs used in this study are listed in Supplementary Tables 2 and 4.

### Protein complex purification for MS analysis

Protein complexes were isolated from HEK293T clones expressing FLAG-ALKBH5 or FLAG-FTO as described before^51^, with a few exceptions: whole cell extracts were prepared by adding lysis buffer (20 mM Tris-HCl (pH 8.0), 137 mM NaCl, 1 mM EDTA, 1% Triton X-100, 10% glycerol, 1.5 mM MgCl_2_, 0.2 mM PMSF, 0.5 mM dithiothreitol (DTT) and 1× protease inhibitor cocktail) directly to the cells, incubating on ice for 15 min and centrifuging lysates at 20,000 × *g* for 15 min in a microcentrifuge at 4 °C. Protein complexes were isolated as described before^51^, with a few exceptions: BC100 buffer (20 mM Tris-HCl (pH 7.8), 100 mM KCl, 0.2 mM EDTA, 10% glycerol, 10 mM β-mercaptoethanol (pH 7.8), 0.2% NP40), was used for all the washing steps instead of BC500 buffer. Protein samples were separated by SDS-PAGE and Coomassie-stained gel was submitted to the Taplin Mass Spectrometry Facility at Harvard Medical School for protein identification in gel sections. The gel bands were cut into approximately 1 mm^3^ pieces. The samples were reduced with 1 mM DTT for 30 min at 60 °C and then alkylated with 5 mM iodoacetamide for 15 min in the dark at room temperature. Gel pieces were then subjected to in-gel trypsin digestion procedure and then washed and dehydrated with acetonitrile for 10 min. followed by the removal of acetonitrile. Pieces were then completely dried in a speed-vac. Rehydration of the gel pieces was with 50 mM ammonium bicarbonate solution containing 12.5 ng/μl modified sequencing-grade trypsin (Promega, V5111) at 4 °C. Samples were then incubated at 37 °C overnight. Peptides were extracted by removing the ammonium bicarbonate solution, followed by one wash step using 50% acetonitrile/1% formic acid. The extracts were then dried in a speed-vac for 1 h. Samples were reconstituted in 10 µl of solvent A (2.5% acetonitrile, 0.1% formic acid). A nano-scale reverse-phase HPLC capillary column was created by packing 2.6 µm C18 spherical silica beads into a fused silica capillary (100 µm inner diameter; 30 cm length) with a flame-drawn tip. After equilibrating the column, each sample was loaded via a Famos autosampler (LC Packings) onto the column. Peptides were eluted using a linear gradient of solvent B (97.5% acetonitrile, 0.1% formic acid). As peptides eluted, they were subjected to electrospray ionization and then entered into an LTQ Orbitrap Velos Pro ion-trap mass spectrometer (ThermoFisher). Eluting peptides were detected, isolated, and fragmented to produce a tandem mass spectrum of specific fragment ions for each peptide. Peptide sequences (and hence protein identity) were determined by matching the human proteome database with the acquired fragmentation pattern by the software program Sequest (ThermoFisher). All databases include a reversed version of all the sequences and the data was filtered with a cutoff at 1% peptide false discovery rate.

### Co-immunoprecipitation

HeLa cells with overexpressed Flag-tag proteins were homogenized in 1 ml of lysis buffer (50 mM Tris-HCl, pH7.5, 150 mM NaCl, 2 mM EDTA, 1% NP-40, cOmplete™ EDTA-Free Protease Inhibitor Cocktail) for 15 min on ice. Cells were further lysed by sonication with low energy (Bioruptor 30 s, on; 30 s off; 6 cycles). The supernatant was collected after centrifugation (14,000 *g*, 10 min). A total of 50 μl of lysate was saved as input, 500 μl of lysate was incubated with 30 ul of anti-Flag M2 magnetic beads overnight at 4 °C with rotation. After washing with lysis buffer for 5 times, the proteins were eluted in 1× LDS loading buffer and resolved on SDS–polyacrylamide gels and detected by western blot analysis.

### m^6^A RNA and caRNA immunoprecipitation and sequencing

1 μg of PolyA+ RNAs and caRNAs was fragmented into ~150 nt by RNA Fragmentation reagent (Ambion, AM8740), and then incubated with 5 μg of anti-m^6^A antibody (Abcam, ab151230) in 500 μl of IP buffer (50 mM Tris, pH 7.4, 100 mM NaCl, 0.05% NP-40 and RNase inhibitor) at 4 °C for 4 h. Thirty microlitres of magnetic protein A beads (Thermo) were added to samples and incubated at 4 °C for 2 h. Beads were washed twice with high-salt buffer (50 mM Tris, pH 7.4, 1 M NaCl, 1 mM EDTA, 1% NP-40, 0.1% SDS), twice with IP buffer, and once with PNK wash buffer (20 mM Tris, pH 7.4, 10 mM MgCl_2_, 0.2% Tween 20). After the PNK treatment, RNA was purified and subjected to library construction by SMARTer smRNA-Seq Kit for Illumina (Clontech).

### eCLIP-seq

METTL3 eCLIP was performed as described previously^52^. In brief, UV crosslinked HeLa cells were collected and resuspended in 1 ml of lysis buffer (50 mM Tris-HCl pH 7.4, 100 mM NaCl, 1% NP-40, 0.1% SDS, 0.5% sodium deoxycholate, cOmplete™ EDTA-Free Protease Inhibitor Cocktail (Roche, 11873580001) and 10 μl of Murine RNase inhibitor (NEB, M0314L)) for 15 min on ice and then sonicated with low energy (Bioruptor 30 s, on; 30 s off; 6 cycles). After RNase-I (Ambion, AM2294) and DNase digestion for 5 min at 37 °C, lysates were centrifuged at 13,000 × *g* for 15 min. Lysates were incubated with antibody-coupled sheep anti-rabbit IgG magnetic beads (30 μl, ThermoFisher, 11203D) at 4 °C overnight. Two percent of lysate was saved size-matched input. Immunoprecipitated (IP) samples were washed with high salt buffer (50 mM Tris-HCl pH 7.4, 1 M NaCl, 1 mM EDTA, 1% NP-40, 0.1% SDS, 0.5% sodium deoxycholate in nuclease-free water) then with wash buffer (20 mM Tris-HCl pH 7.4, 10 mM MgCl_2_, 0.2% Tween 20, in nuclease-free H_2_O). 5′ and 3′ RNA ends were repaired with FastAP (Fermentas, EF0651) and T4 PNK (NEB, M0201L), followed by RNA adapter ligation. Protein-RNA complexes were run on SDS-PAGE gels and transferred to nitrocellulose membranes, and RNA was isolated from the membranes with proteinase K digestion at 37 °C for 20 min then 50 °C for 20 min with interval mixing at 1200 rpm. RNA was cleaned and concentrated using Zymo RNA Clean & Concentrator kit (Zymo Research, R1015) and was reverse transcribed with AffinityScript enzyme (Agilent, 600107) at 54 °C for 20 min. After cDNA end repair, a 3’ ssDNA adapter was ligated. Libraries were amplified according to the Ct values obtained. PCR conditions consisted of 98 °C (30 s) followed by 6 cycles of (98 °C (15 s), 70 °C (30 s), 72 °C (40 s), then (Ct-5) cycles of (98 °C (15 s), 72 °C (45 s) and 72 °C (1 min). Libraries were loaded into a 3% agarose gel and regions between 175–350 bp were extracted and purified using MinElute Gel Extraction Kit (Qiagen, 28604). The quantity and quality of the final libraries were assessed using a Bioanalyzer (Agilent Technology Inc). All samples were multiplexed and sequenced by dual indexed run (PE150) on the Illumina NovaSeq 6000 sequencer. Two biological replicates were conducted for each experiment.

### Cross-Linking Immunoprecipitation (CLIP)

UV crosslinked HeLa cells transfected with reporter constructs were collected and resuspended in 1 ml of lysis buffer (50 mM Tris-HCl pH 7.4, 100 mM NaCl, 1% NP-40, 0.1% SDS, 0.5% sodium deoxycholate, cOmplete™ EDTA-Free Protease Inhibitor Cocktail (Roche, 11873580001) and 10 μl of Murine RNase inhibitor (NEB, M0314L)) for 15 min on ice and then sonicated with low energy (Bioruptor 30 s, on; 30 s off; 6 cycles). Lysates were centrifuged at 13,000 × *g* for 15 min at 4 °C. 10% percent of lysate was saved as input. The remaining lysates were incubated with antibody-coupled sheep anti-rabbit IgG magnetic beads (30 μl, ThermoFisher, 11203D) at 4 °C overnight. Immunoprecipitated (IP) samples were washed with high salt buffer (50 mM Tris-HCl pH 7.4, 1 M NaCl, 1 mM EDTA, 1% NP-40, 0.1% SDS, 0.5% sodium deoxycholate in nuclease free water) then with wash buffer (20 mM Tris-HCl pH 7.4, 10 mM MgCl_2_, 0.2% Tween 20, in nuclease-free H_2_O). IP RNA was isolated from the beads with proteinase K (NEB, P8107S) digestion at 37 °C for 20 min then 50 °C for 20 min with interval mixing at 1200 rpm. RNA was cleaned and concentrated using Zymo RNA Clean & Concentrator kit. RNA was extracted with TRIzol reagent (Invitrogen, 15596018).

### Reverse-transcriptase qPCR

For RT-qPCR, cDNA was generated using Superscript III reverse transcriptase (ThermoFisher, 18080044) according to the manufacturer’s instructions. qPCR assay was performed with Taq Pro Universal SYBR qPCR Master Mix (Vazyme, Q712) according to the manufacturer’s instructions and quantified by the StepOnePlus™ Real-Time PCR System (Applied Biosystem, 4376600). All primers were listed in Supplementary Table 3.

### MeRIP-seq analysis

Raw reads were trimmed using Cutadapt (2.5)^53^ and aligned to hg38 genome using hisat2 (2.1.0)^54^. Peaks were called using exomepeaks (2.16.0)^55^. Transcript GTF file was built for peak identification on caRNA and polyA+ RNA. For differential m^6^A regions of polyA+ RNA upon EIF4A3 depletion, we utilized RADAR software (0.2.4)^44^ with parameters “fragementLength = 150, binSize = 50, minCountsCutOff = 15, cutoff = 0.1, Beta_cutoff = 0.5”. Differential m^6^A regions with |foldchange | >2 were kept. The distribution of the m^6^A peaks was analyzed using MeRIPtools^56^. Peaks were annotated using annotatePeak^57^. Genome coverage bedGraph files were generated by deeptools (3.0.2)^58^ bamCoverage with the parameters “-normalizeUsing RPKM – binSize 5” and visualized using Integrative Genomics Viewer (IGV)^59^. Aggregation plots were generated using deeptools^58^ computeMatrix and plotProfile.

### eCLIP-seq analysis

eCLIP-seq analysis was followed the pipeline^52^. Differential binding peaks were identified using Diffbind (3.6.1)^60^. The distribution of the METTL3 binding peaks was analyzed using deeptools^58^. Genome coverage bedGraph files were generated by deeptools^58^ bamCoverage with the parameters “-normalizeUsing RPKM – binSize 5” and visualized using Integrative Genomics Viewer (IGV)^59^. Aggregation plots were generated using deeptools^58^ computeMatrix and plotProfile.

### mRNA-seq analysis

Raw reads were trimmed using Cutadapt (2.5)^53^ and aligned to hg38 genome using hisat2 (2.1.0)^54^. Differential expression levels of exons and genes were analyzed using featureCounts (2.0.0)^61^ followed by DEseq2 (1.32.0)^62^. Differential expressed exons and genes with |foldchange | >2 were kept. Gene ontology analysis was performed using ClusterProfiler (4.0.5)^63^.

### Statistics and reproducibility

The experiments in this study were performed with three biological replicates and are presented as the mean ± S.D. or mean ± S.E.M. calculated by GraphPad Prism 8.0. Two-tailed unpaired Student’s *t*-test, Mann–Whitney U test, and Fisher’s exact test were performed in GraphPad Prism 8.0 and R Bioconductor. *P*-values <0.05 were considered statistically significant. All statistical tests, resulting *P* values, and numbers of observations are indicated in figure panels or legend.

### Reporting summary

Further information on research design is available in the Nature Portfolio Reporting Summary linked to this article.

## Supplementary information

Supplementary Information

Description of Additional Supplementary Files

Supplementary Data 1

Reporting Summary

## Data Availability

High-throughput sequencing data have been deposited in the Gene Expression Omnibus (GEO) under the accession number GSE207663. The mass spectrometry proteomics data have been deposited to the ProteomeXchange Consortium via the PRIDE partner repository^64^ with the dataset identifier PXD038705. Source data are provided with this paper.

## Source data

Source Data
