# Supplementary material for: Exon junction complex shapes the m6A epitranscriptome
Source: Nat Commun. 2022 Dec 23;13:7904. doi: 10.1038/s41467-022-35643-1 (PMC9780246; doi:10.1038/s41467-022-35643-1)

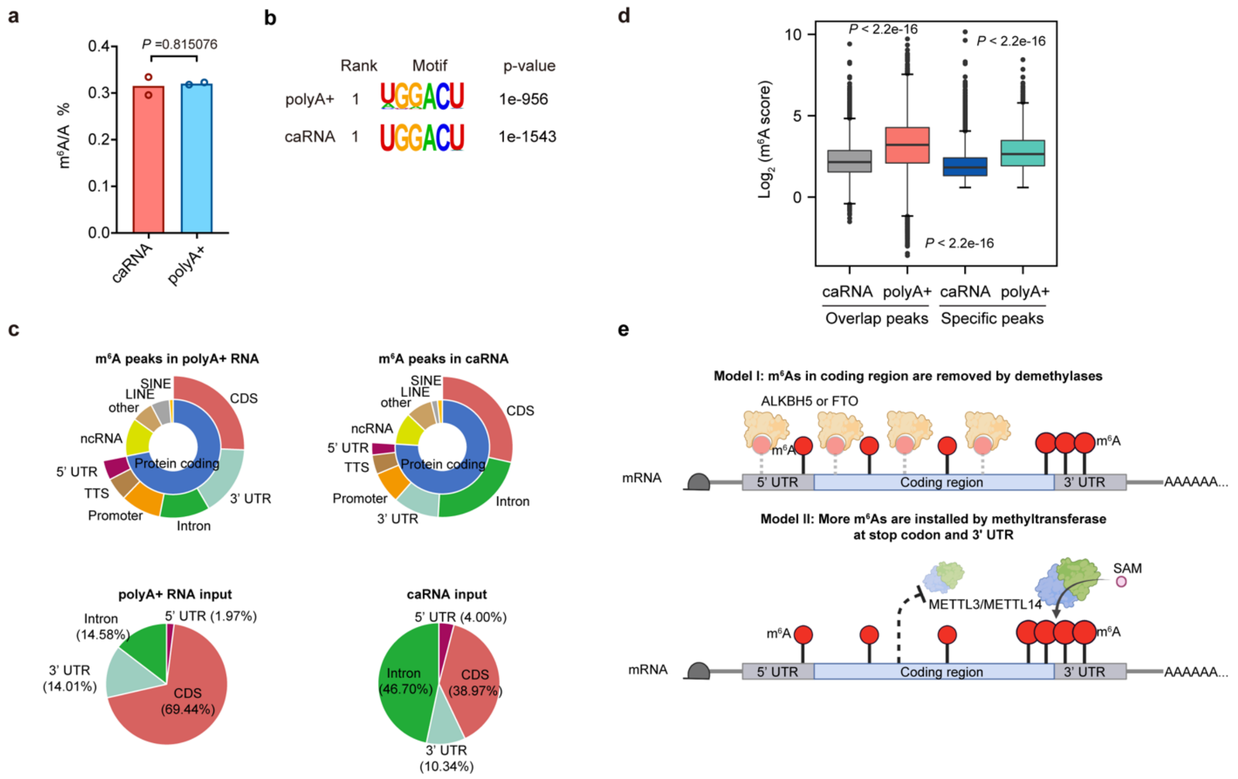

Supplementary Fig. 1. Dynamic m^{6}A modification during mRNA maturation. a. Global m^{6}A levels of caRNA and polyA+ RNA from HeLa cells detected by HPLC-MS/MS. Data are mean ± s.d. Statistics: unpaired, two-tailed t-test. n = 2 independent experiments. Source data are provided as a Source Data file. **b**, Consensus motif of m^{6}A sites in caRNA and polyA+ RNA identified by HOMER motifs software. **c**, The distribution of m^{6}A peaks (upper) and reads (lower) in caRNA and polyA+ RNA. **d**, m^{6}A enrichment of overlap and specific peaks of caRNA and polyA+ RNA. Solid line represents median, with whiskers indicating minimum and maximum value. n = 9,942; 9,942; 16,758; 7,766. Statistical analyses, unpaired two-tailed Student's t-test. **e**, Possible models contributing to the distinct m^{6}A distribution observed in mature RNA. Created with BioRender.com.

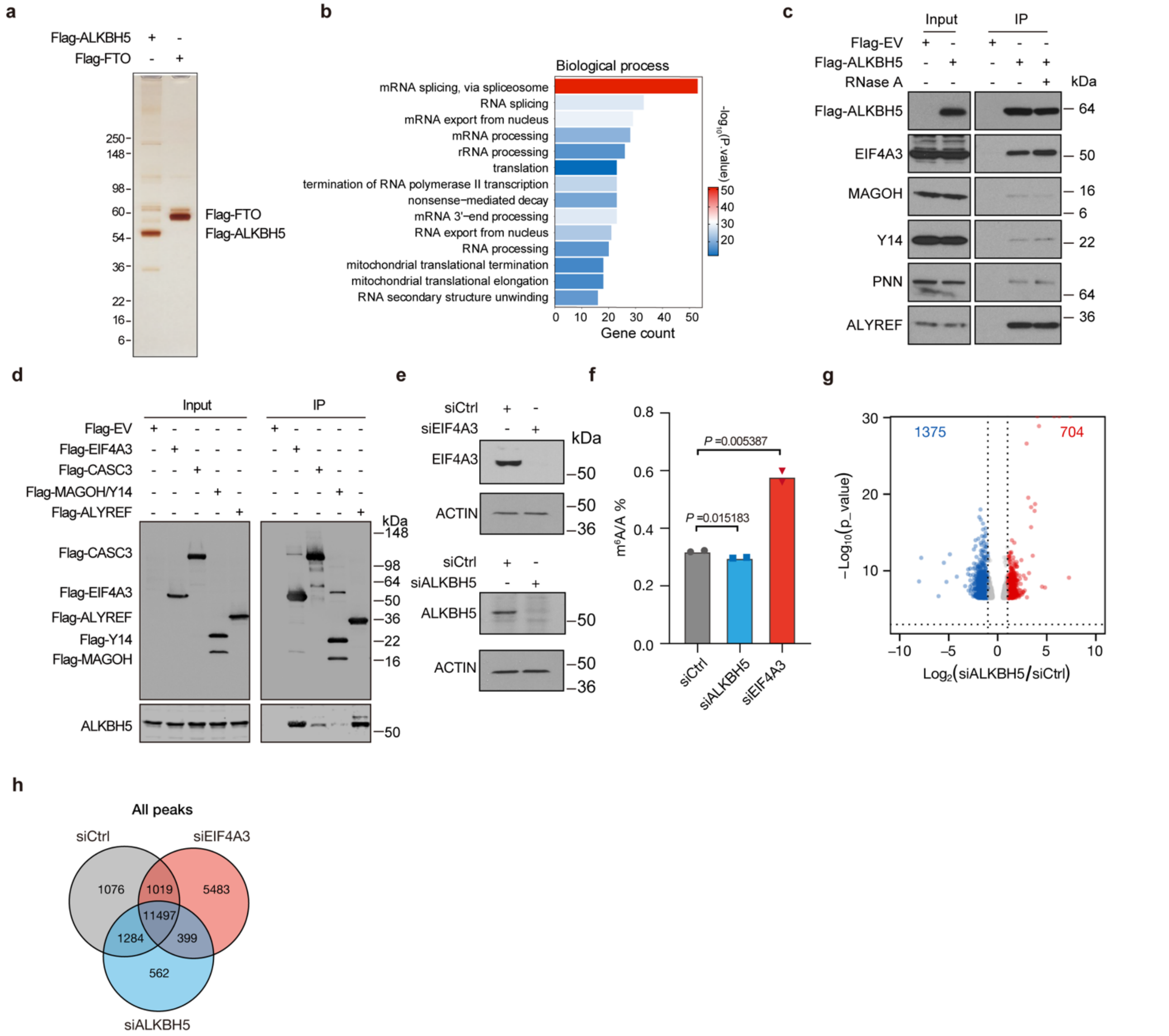

Supplementary Fig. 2. m^{6}A demethylase ALKBH5 does not substantially influence m^{6}A profiles. **a**, Silver staining showing the immunoprecipitation of Flag-ALKBH5 and Flag-FTO. Two independent experiments were repeated with similar results. **b**, Gene ontology analysis of Flag-ALKBH5 interactome. Additional details for proteins interacted with Flag-ALKBH5 or Flag-FTO are provided in Supplementary Data 1. **c**, Immunoprecipitation validation of Flag-ALKBH5 and endogenous EJC components. Two independent experiments were repeated with similar results. **d**, Immunoprecipitation of Flag-tagged EJC component proteins and endogenous ALKBH5. Two independent experiments were repeated with similar results. **e**, West-blot showing the knockdown efficiency of EIF4A3 and ALKBH5. ACTIN is used as the loading control. Two independent experiments were repeated with similar results. **f**, Global m^{6}A

levels of polyA+ RNA from siCtrl, siEIF4A3, and siALKBH5 HeLa cells detected by HPLC-MS/MS. Data are mean ± s.d. Statistics: unpaired, two-tailed t-test. n = 2 independent experiments. Source data are provided as a Source Data file. **g**, Volcano plot showing the differential m^6A regions upon ALKBH5 depletion. **h**, Venn diagram showing the overlap of m^6A peaks identified in polyA+ RNA from siCtrl, siEIF4A3, and siALKBH5 HeLa cells.

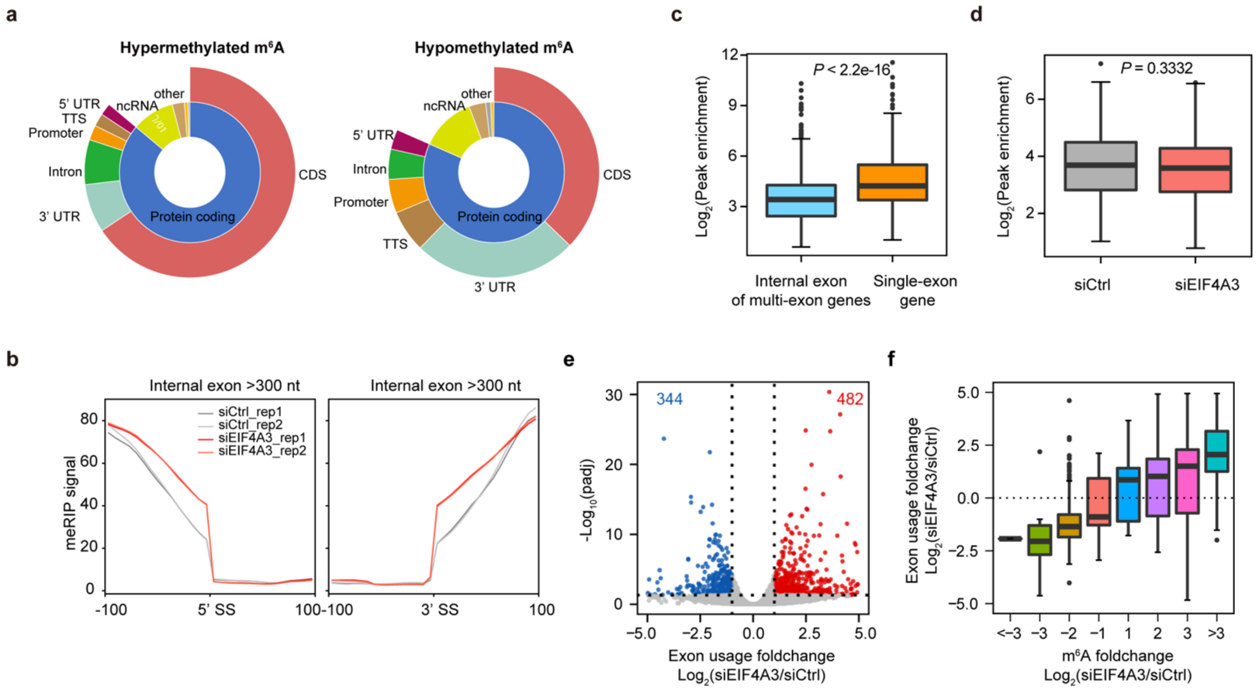

Supplementary Fig. 3. EIF4A3 depletion increases m^{6}A modification near splice junctions and promotes exon inclusion. a, Distribution of hypermethylated m^{6}A and hypomethylated m^{6}A upon EIF4A3 depletion. **b**, Aggregation plots showing m^{6}A enrichment near splice junctions of long internal exons. **c**, The m^{6}A peak enrichment of internal exons of multi-exon genes and single-exon genes. Solid line represents median, with whiskers indicating minimum and maximum value. n = 1,279; 279. Statistical analyses, unpaired two-tailed Student's t-test. **d**, The m^{6}A peak enrichment of single-exon genes in siCtrl (grey) and siEIF4A3 (red). Solid line represents median, with whiskers indicating minimum and maximum value. n = 161; 148. Statistical analyses, unpaired two-tailed Student's t-test. **e**, Differential exon usage upon EIF4A3 depletion. Red dots represent upregulated exons; Blue dots represent downregulated exons. **f**, Positive correlation between m^{6}A foldchange and exon usage fold-change upon EIF4A3 depletion. Solid line represents median, with whiskers indicating minimum to maximum value. n = 1; 9; 91; 34; 26; 169; 124; 64. Statistical analyses, unpaired two-tailed Student's t-test.

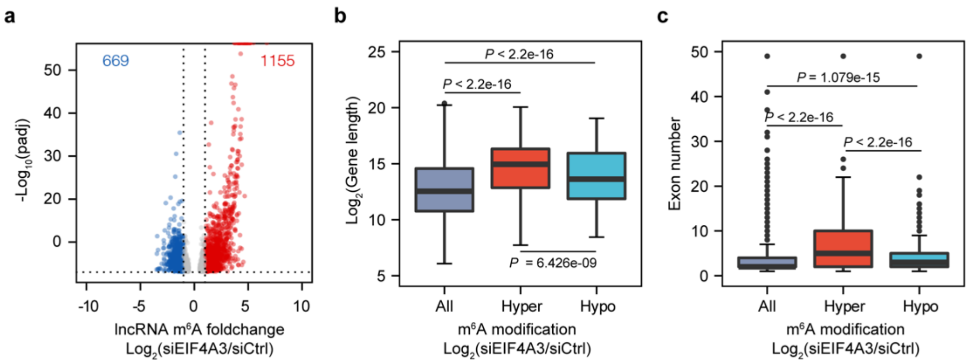

Supplementary Fig. 4. Depletion of EIF4A3 increases m^6A modification of lncRNAs. **a**. Differential m^6A modifications on lncRNAs upon EIF4A3 depletion. R Red dots represent increased m^6A regions (hypermethylated m^6A), Blue dots present decreased m^6A regions (hypomethylated m^6A). **b**. The length of all, hypermethylated or hypomethylated lncRNAs. Solid line represents median, with whiskers indicating minimum to maximum values. n = 17,740; 1,155; 669. **c**. The exon number of all, hypermethylated or hypomethylated lncRNAs. Solid line represents median, with whiskers indicating minimum to maximum values. n = 17,740; 1,155; 669. **d**. Boxplot showing the expression foldchange of lncRNAs with unchanged, hypermethylated, and hypomethylated m^6A regions. Solid line represents median, with whiskers indicating minimum to maximum values. Statistical analyses, unpaired two-tailed Student's t-test.

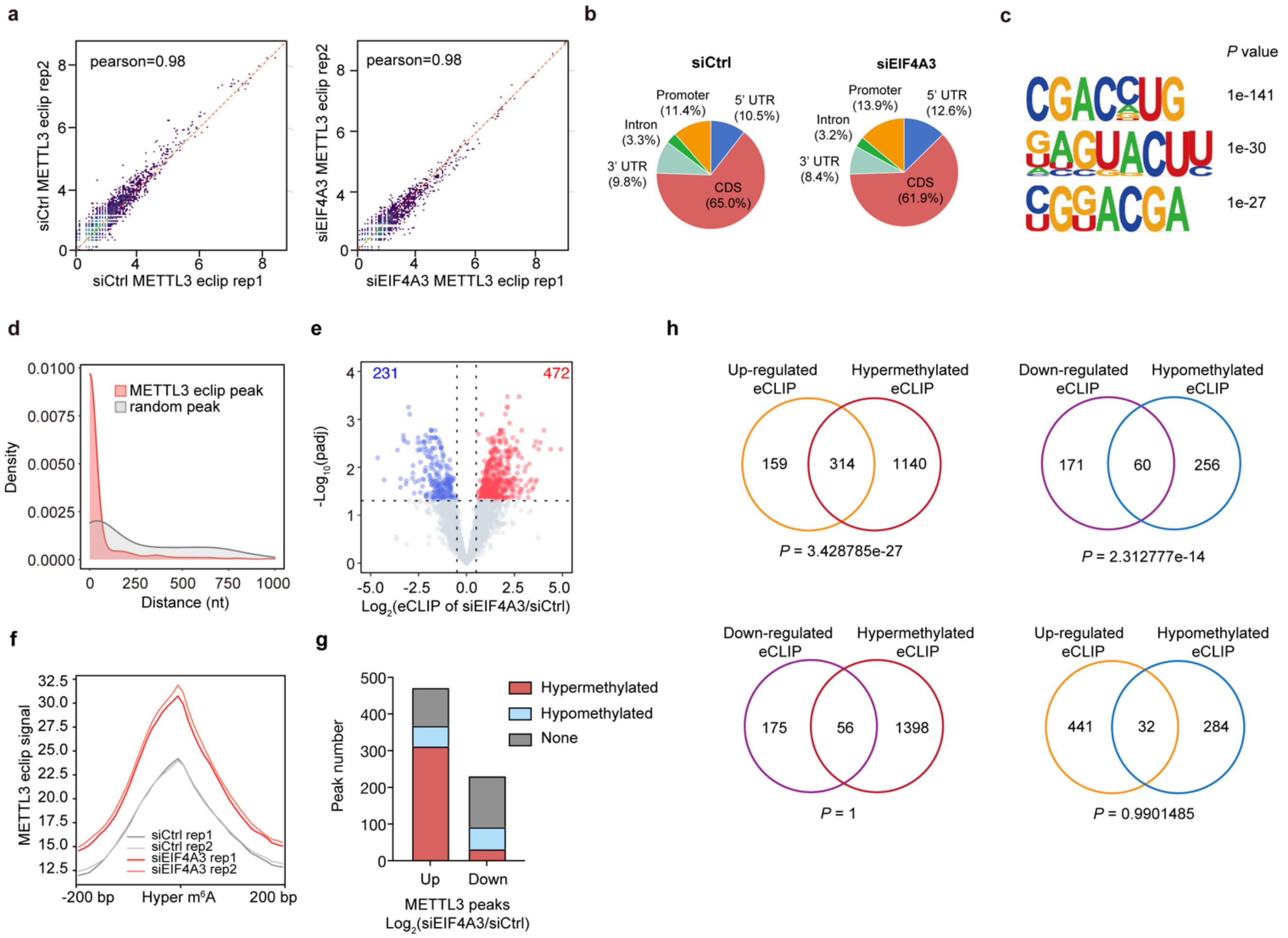

Supplementary Fig. 5. EIF4A3 inhibits METTL3 binding. a, Scatterplot showing the high reproducibility of METTL3 eCLIP-seq in siCtrl and siEIF4A3 cells. **b**, METTL3 binding peak distribution in siCtrl and siEIF4A3 cells. **c**, Top consensus motifs of METTL3 binding peaks identified by HOMER motifs software. **d**, Distribution of distance between m^{6}A sites and METTL3 binding peaks or random peaks. **e**, Differential METTL3 binding peaks upon EIF4A3 depletion. **f**, Average distribution of METTL3 eCLIP-seq signal is shown, aligned around the center of hypermethylated m^{6}A. **g**. The number of increased and decreased METTL3 peaks with hypermethylation or hypomethylated m^{6}A regions. **h**. Venn plots showing the correlation between differential binding and differential methylated METTL3-eCLIP peaks. *P* values are calculated with hypergeometric test.

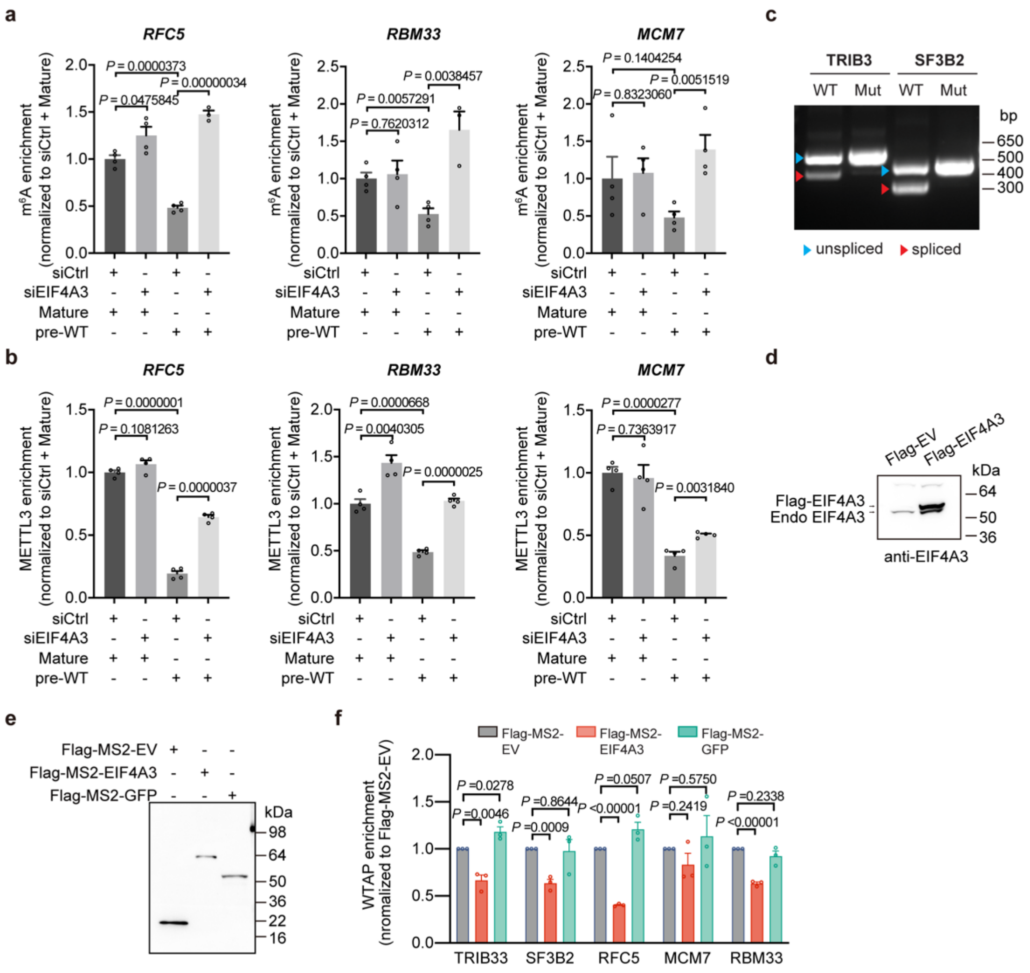

Supplementary Fig. 6. Exon junction complex blocks METTL3-mediated m^{6}A modification of spliced mRNAs. a,**b**, meRIP-qPCR (**a**) and METTL3 CLIP-qPCR (**b**) analysis of reporter constructs showing m^{6}A and METTL3 enrichment upon EIF4A3 KD. Data are mean ± S.E.M. of three independent experiments. Statistical analyses, two-tailed Student's t-test. **c**, analysis of GU/AG splicing defective pre-Mut reporters. Blue triangle indicates the unspliced precursor reporter. Red triangle indicates the spliced reporter. Two independent experiments were repeated with similar results. **d**, Western blotting showing the Flag-tagged and endogenous EIF4A3. Two independent experiments were repeated with similar results. **e**, Western blot showing the expression of Flag-MS2-EV, Flag-MS2-EIF4A3, and Flag-MS2-GFP. Two independent experiments were repeated with similar results. **f**, WTAP CLIP-qPCR analysis of tethering reporter constructs showing WTAP enrichment upon MS2 tagged EV, EIF4A3 or

GFP overexpression. Data are mean ± S.E.M. of three independent experiments. Statistical analyses, two-tailed Student's t-test. Source data are provided as a Source Data file.

Supplementary Table 1. Splicing related proteins interacted with ALKBH5

Complex	Class / family	Gene Symbol	Peptide of ALKBH5 IP	Peptide of FTO IP
spliceosome	PRP19 related	XAB2	20	0
	PRP19 related	SNW1	11	0
	Second step factors	DHX8	14	0
	U1 snRNP	SNRNP70	7	0
	U2 snRNP	SF3A3	6	0
	U4/U6	SART3	20	0
	U4/U6 snRNP	PRPF4B	14	0
	U4/U6 snRNP	PRPF4	14	0
	U4/U6 snRNP	PRPF3	13	0
	U5 snRNP	SNRNP40	6	0
	U5 snRNP	DDX23	10	0
		AQR	28	0
		PLRG1	6	0
		CRNKL1	8	0
		PPIE	7	0
		CWC22	10	0
		DHX16	13	0
		CDC40	6	0
		DDX41	6	0
		CACTIN	6	0
		SLU7	7	0
	U2AF65 associated	DDX39B	2	0
	alternative splicing factor	PTBP1	7	0
EJC/mRNP	EJC/mRNP	RBM8A	26	2
	EJC/mRNP	EIF4A3	120	0
	EJC/mRNP	CASC3	89	0
	EJC/mRNP	PNN	129	0
	EJC/mRNP	ALYREF	262	3
	EJC/mRNP	MAGOHB	48	0
	EJC/mRNP	RNPS1	22	0
hnRNP	hnRNP	RALY	10	0
	hnRNP	HNRNPA1	43	0
	hnRNP	HNRNPA0	8	0
	hnRNP	HNRNPA3	8	0
	hnRNP	HNRNPM	79	0
	hnRNP	HNRNPA2B1	27	2
	hnRNP	HNRNPC	36	0
	hnRNP	RBMX (HNRNPG)	18	1

Supplementary Table 1. Splicing related proteins interacted with ALKBH5 (continued)

Complex	Class / family	Gene Symbol	Peptide of ALKBH5 IP	Peptide of FTO IP
3' end processing	CPSF	CPSF2	6	0
	pre-mRNA/mRNA binding proteins	PABPN1	30	1
	pre-mRNA 3'-end-processing factor	FIP1L1	8	0
		PABPC1	107	5
SR protein	SR protein	SRSF4	16	0
	SR protein	SRSF9	8	0
	SR protein	SRSF1	25	0
	SR protein	TRA2B	12	0
	SR protein	TRA2A	7	0
	SR protein	SRSF10	11	0
	SR related	SRRM1	60	0
others		NCBP1	21	0
		SRRT	10	0
		ZCCHC8	14	0
		DDX5	9	0
		UPF3B	16	0

Supplementary Table 2. Primers for plasmid construction.

Primer name	Primer sequence 5' - 3'
NotI-ALKBH5-f	aacaaGCGGCCGCCAGCGGCTAC
EcoRI-ALKBH5-r	aacaagatatcTCAGTGCCGCCGCATCTTCACCTTTCGGGCAGGG CTGCCTGCTGCCTCAGAGC
ClaI-EIF4A3-f	aacaaATCGATaGCGACCACGGCCACGATG
XbaI-EIF4A3-r	aacaaTCTAGAaTCAGATAAGATCAGCAACGTTCATCGGCATCT C
ClaI-MAGOH-f	aacaaATCGATaGAGAGTGACTTTTATCTGCGTTACTAC
XbaI-MAGOH-r	aacaaTCTAGACTAGATTGGTTTAATCTTGAAGTGTAATCCA
ClaI-Y14-f	aacaaATCGATaGCGGACGTGCTAGATCTTCACGAGGCTG
XbaI-Y14-r	aacaaTCTAGAaTCAGCGACGTCTCCGGTCTGGACTTC
ClaI-CASC3-f	aacaaATCGATaGCGGACCGGCGGCGGCAG
XbaI-CASC3-r	aacaaTCTAGATTAACTGGAACCCCTGCTTACAACCTCAGGTG GAGGGGGCTTGATG
ClaI-ALYREF-f	aacaaATCGATaCCCGATTCCGCGCCCGCCATGG
XbaI-ALYREF-r	aacaaTCTAGATTAACTGGTGTCCATTCTCGCATTATAGGCGTC CAGCTGGGCATCC
Globin_SalI_insert_f	gtcgacGCTTCCCCACCACCAAGA
pre_Globin_SalI_insert_r	GCTAGCAAACATCCTGGGAGA
Globin_SalI_insert_r	GCTAGCAAACATCCTTTCCAG
pre_Globin_HindIII_insert_f	aagcttGTCAACTTCAAGGTATGCGCTGG
Globin_HindIII_insert_r	GGGATCCACACGCAGCTT
Globin_HindIII_insert_f	aagcttGTCAACTTCAAGCTCCTGAGC
RFC5-exon9-f	GCGTCGACAGCACCAATATGGCCTTTG
RFC5-exon9-r	CCCAAGCTTTTCTGTAGGCTGTGGTGAA
TRIB3-exon2-f	GCGTCGACATGCGAGCCACCCCTC
TRIB3-exon2-r	CCCAAGCTTCTTGCAGGTATACTCAGTGCC
MCM7-exon11-f	GCGTCGACGCCAGTACACAACAGGCC
MCM7-exon11-r	CCCAAGCTTCGTAGGTCATTGTCTCGG
SNRPA-exon3-f	GCGTCGACCGTATCCAGTATGCCAAGACC
SNRPA-exon3-r	CCCAAGCTTCGGGACAGGCCCCTG
RBM33-exon9-f	GCGTCGACACTCATTCTCCAAGGTTAATTCCT
RBM33-exon9-r	CCCAAGCTTCTTGAACAGGCGTGACCA
SF3B2-exon10-f	GCGTCGACAAAAACCGGAAGCGTAGG
SF3B2-exon10-r	CCCAAGCTTCTTAAAAGCCTCAAAGATCCTCT
reporter_MS2_insert_f	actctagaaaacatgaggatcacccatgtTGGAAAGGATGTTTGCTAG
reporter_MS2_insert_r	cgacctgcagacatgggtgatcctcatgtGGGCTTCAGCTCCATATTC
NotI-MS2-tag-f	GCGGCCGCAGCTTCTAACTTTACTCAGTTCG
ClaI-MS2-tag-r	GCTATCGATTTGCCGGAGTTTGCTGCG
intron1_GU2AC-f	CCCTGGAAAGacGAGAACAGGAC
intron1_GU2AC-r	gtcctgttctcgtctttccaggg

intron1_AG2TC-f	TCCTTCTCCCtcGATGTTTGCTAG
intron1_AG2TC-r	CTAGCAAACATCgaGGGAGAAGGA
intron2_GU2AC-f	CAACTTCAAGacATGCGCTGGG
intron2_GU2AC-r	CCCAGCGCATgtCTTGAAGTTG
intron2_AG2TC-f	TTGTCTCCGCtcCTCCTGAGCCAC
intron2_AG2TC-r	GTGGCTCAGGAGgaGCGGAGACAA

Supplementary Table 3. Primers for qPCR assays.

Primer name	Primer sequence 5' - 3'
RFC5-endo-f	CAGTGGAGACATGCGTAGGG
RFC5-endo-r	AATGTCTGACTTGAGCGGGT
TRIB3-endo-f	CAGATCGTGCAACTGCTGTG
TRIB3-endo-r	GTACCAGCCAGGACCTCAGT
MCM7-endo-f	ATCCGGGGCAACATCAACAT
MCM7-endo-r	GGTCAGTTCTCCACTCACGG
RBM33-endo-f	ACGACCAATCTGGAGAACAGG
RBM33-endo-r	CTGGGGAGGCATGTGCATT
SF3B2-endo-f	TCAGCGTCAGAGACTGAGGA
SF3B2-endo-r	CCGGGTTGAGTCTTTCTCCC
SNRPA-endo-f	CTCCATGCAGGGTTTCCCTT
SNRPA-endo-r	CTCCACGAAGGTGCCTTTCA
PHGDH-endo-f	TGCGGAAAGTGCTCATCAGT
PHGDH-endo-r	CCCACCACCTGGAGTTTCTC
HCFC1-endo-f	CACCACCCTCATGGTAACGG
HCFC1-endo-r	TCCTGCTGTGTCAGCACAAT
Globin_f	cgtcagatccgctaggatggtgctctctggggaag
RFC5-exon9-r	CCCAAGCTTTTCTGTAGGCTGTGGTGAA
TRIB3-exon2-r	CCCAAGCTTCTTGCAGGTATACTCAGTGCC
MCM7-exon11-r	CCCAAGCTTCGTAGGTCATTGTCTCGG
SNRPA-exon3-r	CCCAAGCTTCGGGACAGGCCCCTG
RBM33-exon9-r	CCCAAGCTTCTTGAACAGGCGTGACCA
SF3B2-exon10-r	CCCAAGCTTCTTAAAAGCCTCAAAGATCCTCT

Supplementary Table 4. siRNA sequence.

Targeted gene name	Strand	Sequence 5' - 3'
siEIF4A3	sense	CGAGCAAUCAAGCAGAUCA
	antisense	UGAUCUGCUUGAUUGCUCG
siALKBH5#1	sense	CUGAGAACUACUGGCGCAA
	antisense	UUGCGCCAGUAGUUCUCAG
siALKBH5#2	sense	ACAAGUACUUCUUCGGCGA
	antisense	UCGCCGAAAGAAGUACUUGU
siCTRL#1	sense	UGGUUUACAUGUCGACUAA
	antisense	UUAGUCGACAUGUAAACCA
siCTRL#2	sense	UGGUUUACAUGUUGUGUGA
	antisense	UCACACAACAUGUAAACCA
siCTRL#3	sense	UGGUUUACAUGUUUUCUGA
	antisense	UCAGAAAACAUGUAAACCA
siCTRL#4	sense	UGGUUUACAUGUUUUCCUA
	antisense	UAGGAAAACAUGUAAACCA

Unprocessed Scans

Supplementary Fig. 2c

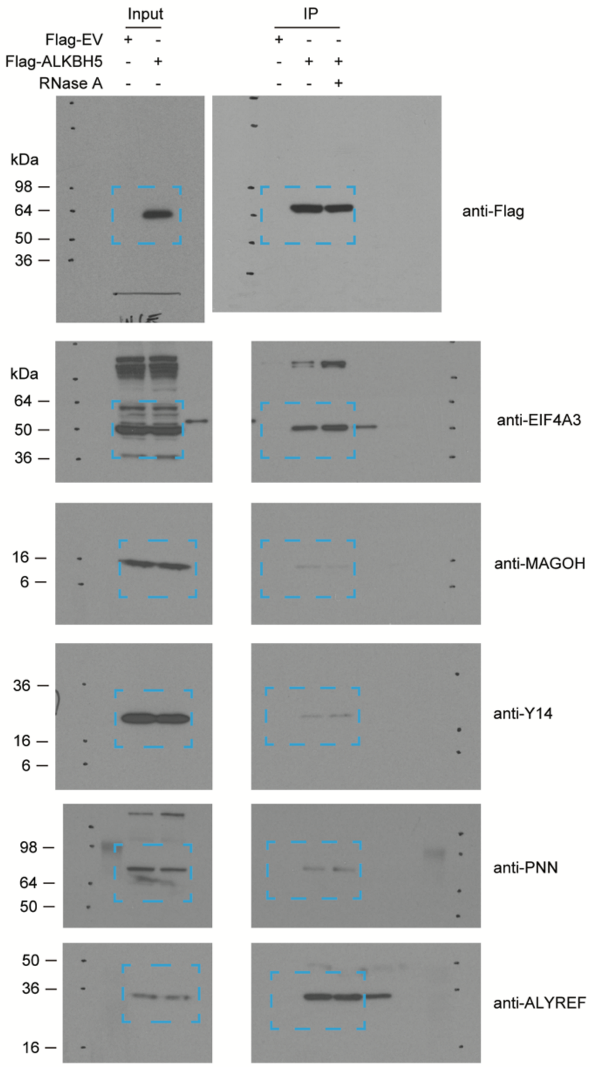

Supplementary Fig. 2d

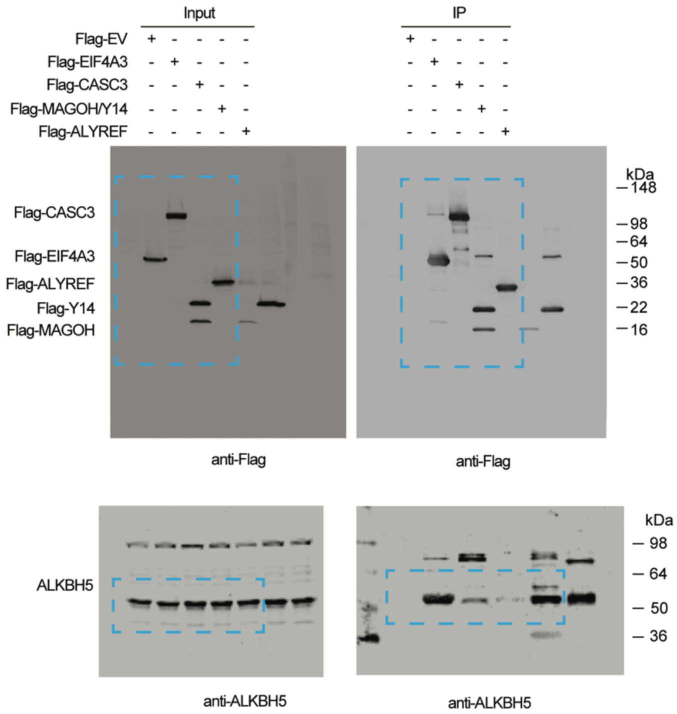

Supplementary Fig. 2e

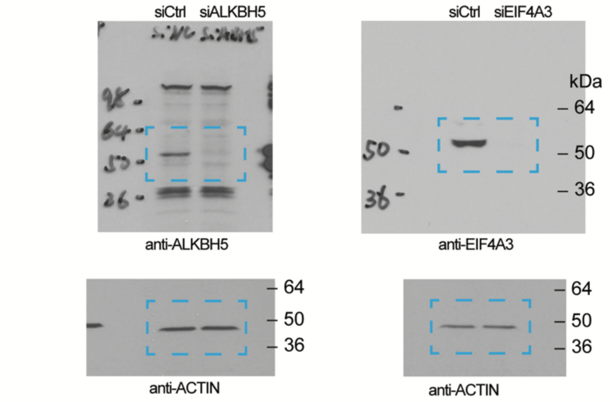

Supplementary Fig. 6c

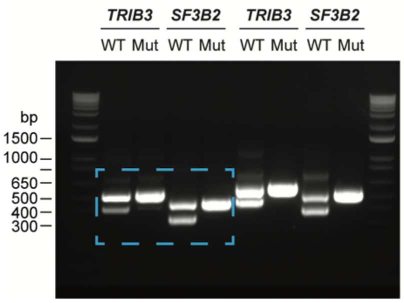

Supplementary Fig. 6d

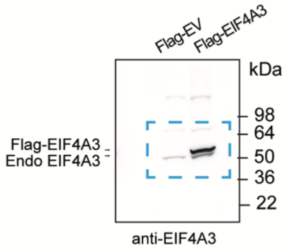

Supplementary Fig. 6e

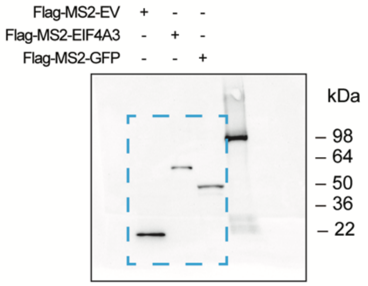

Supplement: Supplementary file 1 — Supplementary Information [file 41467_2022_35643_MOESM1_ESM.pdf]
