# Supplementary material for: Exon junction complex shapes the m6A epitranscriptome
Source: Nat Commun. 2022 Dec 23;13:7904. doi: 10.1038/s41467-022-35643-1 (PMC9780246; doi:10.1038/s41467-022-35643-1)
Supplement: Supplementary file 2 — Description of Additional Supplementary Files [file 41467_2022_35643_MOESM2_ESM.pdf]

Description of Additional Supplementary Files

File Name: Supplementary Data 1
Description: **Mass Spectrometric Analysis of ALKBH5 and FTO interacting proteins**. The list of proteins detected in IP samples from FLAG-ALKBH5, and FLAG-FTO cells. Total peptide number was used to identify potential interacting proteins of ALKBH5 or FTO.
