# Supplementary material for: Exon junction complex shapes the m6A epitranscriptome
Source: Nat Commun. 2022 Dec 23;13:7904. doi: 10.1038/s41467-022-35643-1 (PMC9780246; doi:10.1038/s41467-022-35643-1)
Supplement: Supplementary file 4 — Reporting Summary [file 41467_2022_35643_MOESM4_ESM.pdf]

nature portfolio

Corresponding author(s): Richard I. Gregory

Last updated by author(s): Dec 9, 2022

Reporting Summary

Nature Portfolio wishes to improve the reproducibility of the work that we publish. This form provides structure for consistency and transparency in reporting. For further information on Nature Portfolio policies, see our Editorial Policies and the Editorial Policy Checklist.

Statistics

For all statistical analyses, confirm that the following items are present in the figure legend, table legend, main text, or Methods section.

n/a	Confirmed	
☐	☒	The exact sample size (*n*) for each experimental group/condition, given as a discrete number and unit of measurement
☐	☒	A statement on whether measurements were taken from distinct samples or whether the same sample was measured repeatedly
☐	☒	The statistical test(s) used AND whether they are one- or two-sided<br>*Only common tests should be described solely by name; describe more complex techniques in the Methods section.*
☒	☐	A description of all covariates tested
☒	☐	A description of any assumptions or corrections, such as tests of normality and adjustment for multiple comparisons
☐	☒	A full description of the statistical parameters including central tendency (e.g. means) or other basic estimates (e.g. regression coefficient) AND variation (e.g. standard deviation) or associated estimates of uncertainty (e.g. confidence intervals)
☐	☒	For null hypothesis testing, the test statistic (e.g. *F*, *t*, *r*) with confidence intervals, effect sizes, degrees of freedom and *P* value noted<br>*Give P values as exact values whenever suitable.*
☒	☐	For Bayesian analysis, information on the choice of priors and Markov chain Monte Carlo settings
☒	☐	For hierarchical and complex designs, identification of the appropriate level for tests and full reporting of outcomes
☐	☒	Estimates of effect sizes (e.g. Cohen's *d*, Pearson's *r*), indicating how they were calculated

Our web collection on statistics for biologists contains articles on many of the points above.

Software and code

Policy information about availability of computer code

Data collection	qPCR data was collected by using StepOnePlus™ Real-Time PCR System (Applied Biosystems). High Through-put sequencing data was collected by Illumina Novaseq platform. LC/MS-MS data was collected by using Agilent Mass Hunter LC/MS Data Acquisition Version B.08.0 .
Data analysis	Statistical analysis was performed with GraphPad Prism 8. meRIP-seq and eCLIP-seq analysis used following software: Cutadapt-2.5, hisat2-2.1.0, Exomepeaks-2.16.0, RADAR software-0.2.4, MeRIPtools-0.2.1, annotatePeak, bedtools-2.27.1,samtools-1.9, deeptools-3.0.2, CLIPper (https://github.com/YeoLab/clipper), Diffbind-3.6.1, featureCounts-2.0.0, ClusterProfiler-4.0.5, DEseq2-1.32.0, findMotifsGenome. Plots were generated using R-4.1.1. Quantitative Analysis Version B.07.01 software was used for LC-MS/MS data analysis.

For manuscripts utilizing custom algorithms or software that are central to the research but not yet described in published literature, software must be made available to editors and reviewers. We strongly encourage code deposition in a community repository (e.g. GitHub). See the Nature Portfolio guidelines for submitting code & software for further information.

Data

Policy information about availability of data

All manuscripts must include a data availability statement. This statement should provide the following information, where applicable:

- Accession codes, unique identifiers, or web links for publicly available datasets
- A description of any restrictions on data availability
- For clinical datasets or third party data, please ensure that the statement adheres to our policy

High-throughput sequencing data have been deposited in the Gene Expression Omnibus (GEO) under the accession number GSE207663.
Proteomic data have been submitted to MassIVE under the number MSV000090641.

Human research participants

Policy information about studies involving human research participants and Sex and Gender in Research.

Reporting on sex and gender	N/A
Population characteristics	N/A
Recruitment	N/A
Ethics oversight	N/A

Note that full information on the approval of the study protocol must also be provided in the manuscript.

Field-specific reporting

Please select the one below that is the best fit for your research. If you are not sure, read the appropriate sections before making your selection.

☒ Life sciences ☐ Behavioural & social sciences ☐ Ecological, evolutionary & environmental sciences

For a reference copy of the document with all sections, see nature.com/documents/nr-reporting-summary-flat.pdf

Life sciences study design

All studies must disclose on these points even when the disclosure is negative.

Sample size	For sequencing data, two replicates with at least 30M reads are used for each sample type.<br>For qPCR anaylsis, three replicates are used for each sample type.
Data exclusions	No data was excluded
Replication	All experiments were repeated at least twice, and similar results were observed.
Randomization	Experiments were all randomized.
Blinding	The investigators were blinded to group allocation during data collection and analysis.

Reporting for specific materials, systems and methods

We require information from authors about some types of materials, experimental systems and methods used in many studies. Here, indicate whether each material, system or method listed is relevant to your study. If you are not sure if a list item applies to your research, read the appropriate section before selecting a response.

Materials & experimental systems

n/a	Involved in the study	
☐	☒	Antibodies
☐	☒	Eukaryotic cell lines
☒	☐	Palaeontology and archaeology
☒	☐	Animals and other organisms
☒	☐	Clinical data
☒	☐	Dual use research of concern

Methods

n/a	Involved in the study	
☒	☐	ChIP-seq
☒	☐	Flow cytometry
☒	☐	MRI-based neuroimaging

Antibodies

Antibodies used

Mouse anti-Flag M2 (sigma, F3165), anti-EIF4A3 (Abcam, ab32485), anti-CASC3 (Invitrogen, A302-471A), anti-Y14 (Abcam, ab5828), anti-MAGOH (Abcam, ab186431), anti-PNN (Abcam, 244250), anti-ALKBH5 (Cell Signaling Technology, 80283), anti-ALYREF (Abcam, ab202894), anti-METTL3 (Abcam, ab195352), anti-m6A (Abcam, ab151230), anti-beta-Actin (Abcam, 8226), anti-rabbit IgG, HRP-linked Antibody (Cell Signaling Technology, 7074), anti-mouse IgG, HRP-linked Antibody (Cell Signaling Technology, 7076).

Validation

Mouse monoclonal anti-Flag M2 (sigma, F3165): https://www.sigmaaldrich.com/US/en/product/sigma/f3165. Western blot, and IP commercial validations. Validate in-house by western blotting (1:2000).
Rabbit polyclonal Rabbit anti-EIF4A3 (Abcam, ab32485) : https://www.abcam.com/eif4a3-antibody-ab32485.html. ICC/IF, IHC-P, IP, WB commercial validations. Validate in-house by western blotting (1:2000).
Rabbit polyclonal anti-CASC3 (Invitrogen, A302-471A) : https://www.thermofisher.com/antibody/product/CASC3-Antibody-Polyclonal/A302-471A. IHC WB commercial validations. Validate in-house by western blotting (1:2000).
Mouse monoclonal anti-Y14 (Abcam, ab5828) : https://www.abcam.com/y14-antibody-4c4-ab5828.html. ICC/IF, IHC-P, IP, WB commercial validations. Validate in-house by western blotting (1:2000).
Rabbit monoclonal anti-MAGOH (Abcam, ab180505) : https://www.abcam.com/magoh-antibody-epr14037-ab180505.html. IHC WB commercial validations. Validate in-house by western blotting (1:2000).
Rabbit polyclonal anti-PNN (Abcam, 244250): https://www.abcam.com/pnndrsp-antibody-ab244250.html. ICC/IF, IHC-P, WB commercial validations. Validate in-house by western blotting (1:2000).
Rabbit monoclonal anti-ALKBH5 (Cell Signaling Technology, 80283) : https://www.cellsignal.com/products/primary-antibodies/alkbh5-e5y7c-rabbit-mab/80283. WB commercial validations. Validate in-house by western blotting (1:1000).
Rabbit monoclonal anti-ALYREF (Abcam, ab202894) : https://www.abcam.com/alyref-antibody-epr17942-ab202894.html. IHC-P, WB commercial validations. Validate in-house by western blotting. IHC-P, WB commercial validations. Validate in-house by western blotting (1:2000).
Rabbit monoclonal anti-METTL3 (Abcam, ab195352) : https://www.abcam.com/mettl3-antibody-epr18810-ab195352.html. ICC/IF, IHC-P, IP, WB commercial validations. Validate in-house by western blotting (1:2000) and IP (1:100).
Rabbit polyclonal anti-m6A (Abcam, ab151230) : https://www.abcam.com/n6-methyladenosine-m6a-antibody-ab151230.html. Validate in-house by IP (1:100).
Mouse monoclonal anti-beta-Actin (Abcam, 8226) : https://www.abcam.com/beta-actin-antibody-mabcam-8226-loading-control-ab8226.html. ICC/IF, WB commercial validations. Validate in-house by western blotting (1:3000).
Goat anti-rabbit IgG, HRP-linked Antibody (Cell Signaling Technology, 7074) : https://www.cellsignal.com/products/secondary-antibodies/anti-rabbit-igg-hrp-linked-antibody/7074?_=1658526081877&Ntt=7074&tahead=true. WB commercial validations. Validate in-house by western blotting (1:3000).
Goat anti-mouse IgG, HRP-linked Antibody (Cell Signaling Technology, 7076) : https://www.cellsignal.com/product/productDetail.jsp?productId=7076. WB commercial validations. Validate in-house by western blotting (1:3000).
Mouse monoclonal anti-WTAP (Proteintech, 60188): https://www.ptglab.com/products/WTAP-Antibody-60188-1-Ig.htm. WB, IP, IHC, IF, FC, ELISA. commercial validations. Validate in-house by western blotting (1:2000) and IP (1:100).

Eukaryotic cell lines

Policy information about cell lines and Sex and Gender in Research

Cell line source(s)	All cell lines used in this study, including HEK293T and HeLa, were purchased from ATCC.
Authentication	Cell lines were authenticated with morphology, karyotyping, and PCR based approaches by ATCC
Mycoplasma contamination	All cell lines were tested negative for mycoplasma contamination by the sequencing data.
Commonly misidentified lines (See ICLAC register)	No commonly misidentified cell lines were used.
